# Evolving Landscape of Glioblastoma Research: Integrating Therapeutic Advances and Diagnostic Frontiers

**DOI:** 10.3390/brainsci16050487

**Published:** 2026-04-30

**Authors:** Nirupama A. Sabnis, Luke C. Cooksey, Hareesh Jayakumar, Mariana Moguel Mendez, Ezek Mathew, Roland Max Petty, Amalendu Ranjan, Luis Colon-Perez, Rob Dickerman, Porunelloor A. Mathew, Bruce A. Bunnell

**Affiliations:** 1 Department of Microbiology, Immunology and Genetics, University of North Texas Health, Fort Worth, TX 76107, USA; lukecooksey@my.unthsc.edu (L.C.C.); ezekmathew@my.unthsc.edu (E.M.); amalendu.ranjan@unthsc.edu (A.R.); porunelloor.mathew@unthsc.edu (P.A.M.); bbunnell@illinois.edu (B.A.B.); 2 Biology Department, Uppsala University, 753 10 Uppsala, Sweden; hareesh.jayakumar.7259@student.uu.se; 3 Burnette School of Medicine, Texas Christian University, Fort Worth, TX 76109, USA; mlmmendez@outlook.com; 4 College of Science and Engineering, Texas Christian University, Fort Worth, TX 76109, USA; max.petty@tcu.edu; 5 Department of Pharmacology and Neuroscience, University of North Texas Health, Fort Worth, TX 76107, USA; luis.colon-perez@unthsc.edu; 6 Systems Science, University of North Texas Health, Fort Worth, TX 76107, USA; rob.dickerman@unthsc.edu

**Keywords:** glioblastoma (GB), GBM, targeted therapies, biomimetic nanocarriers, immunotherapy, molecular imaging, machine learning, blood–brain barrier, precision medicine

## Abstract

Glioblastoma (GB) remains the most aggressive primary brain malignancy, with the Stupp regimen persisting as the standard of care for nearly two decades despite poor survival outcomes. This review was synthesized by extensively reviewing and analyzing the literature from PubMed, Scopus, and Web of Science to evaluate the emerging promising therapeutic and diagnostic strategies for combating GB. Results indicate significant progress in molecularly targeted therapies, biomimetic nanocarriers, and advanced radiotherapy. While immunotherapeutic approaches, such as checkpoint inhibitors and vaccines, show variable clinical success, the integration of bioinformatics and machine learning has significantly enhanced treatment response prediction. Furthermore, advances in radiomics and molecular imaging have improved the differentiation between true tumor progression and pseudoprogression, potentially reducing invasive diagnostic requirements. Additionally, other emerging and investigational adjuvant therapeutic approaches have shown promise. We conclude that, while multimodal strategies integrating molecular and computational approaches offer a path toward personalized GB management, significant barriers—namely tumor heterogeneity and the blood–brain barrier—persist. Future research must prioritize precision-based combinatorial models to successfully translate these preclinical advancements into improved clinical outcomes for patients.

## 1. Introduction

Glioblastoma (GB) stands as one of the most formidable challenges in the field of neuro-oncology. Its pathological hallmarks include rapid proliferation, heterogeneity, and a tendency for recurrence, as well as microenvironmental immunosuppression and genomic instability, which drive the poor prognosis observed in patients. The complex nature of the disease is evident in its varied cellular composition and intricate molecular biology, making it a moving target in therapeutic terms [1,2,3,4]. Establishing an accurate diagnosis and etiology, selecting the appropriate treatment, and improving the long-term prognosis are critical yet challenging objectives in neurosurgery and cancer research.

### 1.1. Standard of Care and Current Clinical Outcomes

The cornerstone of GB treatment has traditionally been a multi-modal approach. Since its release in 2005, the Stupp regimen has been the gold standard for treating GB and has significantly increased survival rates [5]. It is named after a Swiss oncologist who published his studies in 2005. It comprises surgical resection, radiotherapy, and chemotherapy. Temozolomide (TMZ) has emerged as a standard chemotherapeutic agent, routinely integrated with radiation therapy and surgery to provide the synergistic effects required by the standard clinical protocol. These treatments primarily aim to extend survival and enhance the quality of life for patients, even though they do not offer a cure [6]. Low-intensity alternating electric fields applied via transducer arrays positioned on the patient’s scalp were shown to improve overall survival in primary GB from 16 months to 20.9 months when added to the standard of care (SOC) of tumor treatment fields (TT Fields) [7]. Although the US FDA has approved TT Fields as an adjuvant therapy for newly diagnosed GB, its application in clinical practice has not expanded to date [8,9]. Nonetheless, the average overall survival (OS) period for patients with newly diagnosed GB is still 15 months, and a five-year survival rate is only 7.2% [10]. Despite incremental advances, the current therapeutic landscape for GB is fraught with limitations. The overall survival benefit remains modest at best. The heterogeneity and aggressive nature of the tumor contribute to the challenge, often outpacing the efficacy of conventional treatments. Furthermore, the inability to achieve significant and lasting control over the disease underscores the need for more effective therapeutic strategies [11].

### 1.2. Key Barriers in Therapeutic Efficacy

Major Concerns in GB therapeutics are crossing the blood–brain barrier (BBB), drug resistance and gene heterogeneity of the tumor. In addition, the immunosuppressive microenvironment is one of the key factors for the low success rate of standard therapies.

#### 1.2.1. The Blood–Brain Barrier (BBB)—Biological Constraints and Integrated Bypass Strategies

A significant impediment in the effective treatment of GB is the BBB, which is characterized by highly restrictive tight junctions and polarized efflux transporters (such as P-glycoprotein) that exclude over 98% of small-molecule drugs from the central nervous system (CNS). This is a natural defense mechanism of the brain that limits the passage of substances from the bloodstream, posing a major hurdle for delivering therapeutic agents to the tumor site. Overcoming this barrier is crucial for enhancing the effectiveness of GB treatments and is a major focus of current research [12,13,14,15]. Modern bypass strategies have shifted toward an integrated framework that categorizes interventions by their method of barrier penetration. Nanoformulations offer a biochemical approach, utilizing receptor-mediated transcytosis to shuttle therapeutics across the intact endothelium [11,14]. In contrast, focused ultrasound (FUS) combined with systemic microbubbles provides a localized physical disruption by transiently opening tight junctions, allowing for high-dose delivery to specific coordinates. Alternatively, nose-to-brain delivery provides a physiological bypass by utilizing the olfactory and trigeminal nerve pathways to deliver agents directly to the cerebrospinal fluid (CSF), circumventing the systemic circulation entirely. Recent evidence suggests that the most effective clinical outcomes may arise from “precision delivery” models that combine these strategies, such as using FUS to enhance the penetration of targeted nanocarriers in a synergistic manner.

#### 1.2.2. Molecular and Cellular Drivers of Drug Resistance

The profound drug resistance observed in GB is primarily driven by a combination of unique physiological barriers and intrinsic cellular plasticity. Within the tumor itself, a subpopulation of glioblastoma stem cells (GSCs) exhibits heightened DNA-repair mechanisms and overexpresses efflux pumps, such as P-glycoprotein, which actively expel chemotherapeutic agents like TMZ. Furthermore, the high degree of inter- and intra-tumoral heterogeneity allows the tumor to bypass targeted therapies by activating alternative signaling pathways or modulating the immune-suppressive microenvironment. This multifaceted resistance necessitates the development of advanced therapeutic modalities of bypassing these defenses to achieve effective therapeutic concentrations at the tumor site [2,3,11].

#### 1.2.3. Gene Tumor Heterogeneity

GB is often characterized by profound inter-tumoral and intra-tumoral heterogeneity, where a single tumor mass can contain multiple distinct molecular subtypes, such as proneural, mesenchymal, and classical. This genomic diversity is driven by a chaotic landscape of chromosomal alterations, which vary significantly even between neighboring cells. Such heterogeneity allows subclonal populations to survive therapeutic pressure, as cells lacking a specific drug target can continue to proliferate and drive recurrence. Consequently, this genetic mosaicism is a primary reason why single-agent targeted therapies often fail, necessitating more complex multi-modal treatment strategies [3,4]. 

#### 1.2.4. Immunosuppressive Microenvironment

The GB microenvironment is characterized by a “cold” immune landscape, where the tumor actively recruits myeloid-derived suppressor cells (MDSCs) and regulatory T cells (Tregs) to stifle effective immune responses. Tumor cells and associated astrocytes secrete potent immunosuppressive cytokines, such as transforming growth factor-beta (TGF-ß) and Interleukin-10 (IL-10), which polarize resident microglia into a pro-tumorigenic M2 phenotype. Furthermore, the upregulation of immune checkpoints like programmed death-ligand 1 (PD-L1) creates a physical and chemical shield that induces T cell exhaustion, rendering standard immunotherapies largely ineffective [15,16].

**Figure 1:**
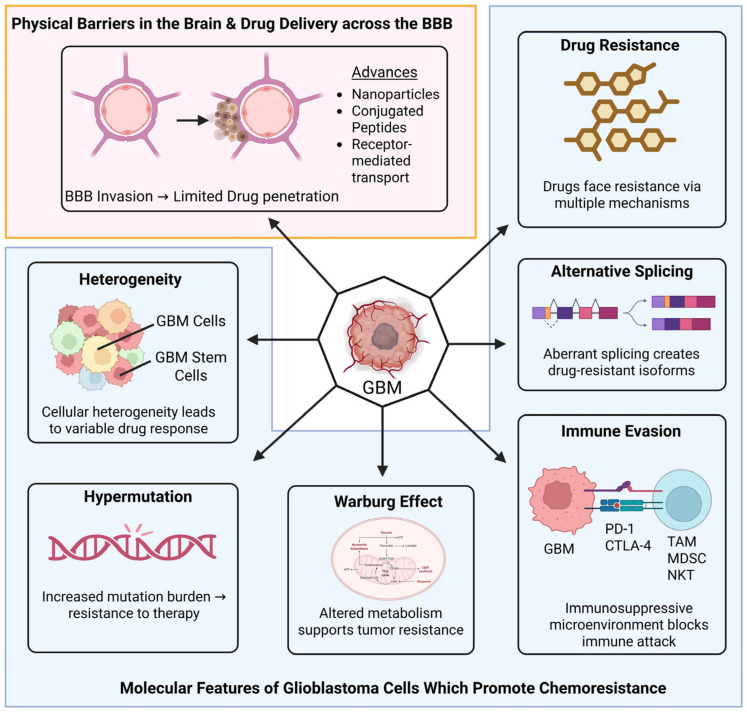
Schematic representation of major gridlocks in glioblastoma therapeutics. (Created in BioRender. Ranjan, A. (2026) https://BioRender.com/ps8vh5y).

Figure 1 captures the major gridlocks in GB therapeutics. The lack of treatment specificity not only harms adjacent healthy brain tissue, leading to serious side effects and premature treatment discontinuation, but also fails to address the inherent biological defenses of the tumor. Beyond these toxicity concerns, understanding and overcoming these mechanisms of resistance are critical for developing more effective and durable treatment modalities [11,16,17].

The diagram provides a comprehensive overview of the physiological, molecular, and immunological barriers that collectively contribute to the high failure rate of glioblastoma (GB) treatments. (A) Physical Barriers: The blood–brain barrier (BBB) is highlighted as the primary gatekeeper, where tight junctions and efflux transporters severely restrict the intracranial penetration of systemic chemotherapies. (B) Internal Resistance Mechanisms: This section illustrates the “moving target” nature of GB, driven by inter- and intra-tumoral heterogeneity. Key features include the presence of glioma stem cells (GSCs) that drive recurrence, metabolic reprogramming (the Warburg effect) that provides a survival advantage in hypoxic conditions, and hypermutation phenotypes that facilitate acquired resistance to alkylating agents. (C) External Environment and Immunosuppression: The schematic details the “cold” tumor microenvironment, where GB cells exploit immune checkpoints (Programmed Cell Death Protein 1 (PD-1), Cytotoxic T-Lymphocyte-Associated Protein 4 (CTLA-4)) and recruit suppressive cell populations. These include Tumor-Associated Macrophages (TAMs) and myeloid-derived suppressor cells (MDSCs), which actively inhibit the cytotoxic activity of Natural Killer (NK) and T cells. Together, these interlocking gridlocks necessitate the development of the multimodal, nanoparticle-based, and adaptive strategies discussed in this review. 

### 1.3. From Neuroimaging to Liquid Biopsy: Enhancing Diagnostic Precision

GB is traditionally diagnosed using neuroimaging techniques like computed tomography (CT) or magnetic resonance imaging (MRI), with postoperative confirmation through histopathology and molecular investigation [18,19,20,21]. Imaging methods have trouble telling the difference between changes brought on by treatment and tumor development, which could cause misunderstandings and delays in treatment. Likewise, tissue biopsies are intrusive and unsuitable for tracking the effects of ongoing treatments, despite their informational value. As a result of these difficulties, liquid biopsy—especially using blood samples—has emerged as a viable substitute for GB diagnosis and surveillance [22,23,24,25]. Currently, a minimally invasive method of gathering tumor-related data to direct treatment is provided via blood and cerebrospinal fluid (CSF) collection. Additionally, tissue biopsies offer more precise insights into tumor morphology and the microenvironment. Therefore, researchers are developing liquid biopsy as a complementary method to enhance the diagnostic accuracy and monitoring of GB patients by providing additional information alongside traditional tissue biopsies. Moreover, utilizing a combination of diverse biomarker types may enhance clinical effectiveness compared to solely relying on one biomarker category, which will potentially improve diagnostic sensitivity and specificity and will address some of the existing limitations associated with liquid biomarkers for GB.

Glioblastoma’s immunosuppressive microenvironment, driven by cells like macrophages and stem cells alongside hypoxic conditions, allows the tumor to evade the immune system and resist standard treatments. These complex factors make the development of effective immunotherapies essential for overcoming the current challenges in treating this aggressive cancer. Also, due to the significant intratumoral phenotype, intrinsic heterogeneity, and widespread infiltration of tumor cells throughout the brain, novel therapeutic approaches are urgently needed. Furthermore, there are limited options for second-line therapies. Similarly, current diagnostic methods used for GB exhibit challenges. Thus, interventions in disease management of GB are urgently needed. The present status of GB research is thoroughly reviewed in this article, which also highlights the various therapeutic strategies and diagnostic plans that scientists are researching and the challenges that researchers and clinicians are encountering in the development of GB theragnostic. It also provides a clinician’s viewpoint on research that may soon be used in therapeutic settings.

## 2. Recent Advances in Molecular Biology and/or GB Biomarkers

### 2.1. New Frontiers in Classification: Differentiating GB from Histological Mimics

The 2021 WHO classification of CNS tumors fundamentally integrated molecular markers with traditional histology to define GB. Under these guidelines, adult GB was defined as IDH-wildtype diffuse astrocytic tumor, often accompanied by specific molecular ‘signatures’ such as Epidermal Growth Factor Receptor (EGFR) amplification, telomerase reverse transcriptase (TERT) promoter mutations, or the concurrent chromosome alterations: the gain of chromosome 7 and loss of chromosome 10 (−7/+10), even in the absence of high-grade histological features [26,27]. However, under the proposed 2026 guidelines, as of April 2026 the term “Glioblastoma” is strictly reserved for IDH-wildtype astrocytic tumors in adults [28]. According to the new revisions, the following changes are made (more changes are on the way):

A. **The “IDH-Mutant GBM” term is no longer recognized:** Tumors that were previously called “IDH-mutant glioblastomas” are now classified as Astrocytoma, IDH-mutant, CNS WHO grade 4.
B. **Molecular vs. Histological Diagnosis:** A tumor can be diagnosed as GB even if it lacks “classic” histological features like necrosis or microvascular proliferation, provided it carries specific molecular markers.
C. **The Essential Diagnostic Criteria:** For a diffuse astrocytic glioma in an adult to be classified as GB, IDH-wildtype (Grade 4), it must be IDH-wildtype and H3-wildtype and meet at least one of the following criteria: molecular TERT-promoter mutations, EGFR gene amplification, or +7/−10 chromosome copy number alterations, allowing for a definitive Grade 4 diagnosis even in the absence of microvascular proliferation or necrosis.
D. **Key Molecular and Genetic Changes:** The 6th Edition places higher emphasis on refined molecular profiling to distinguish GBM from “mimics” that may look identical under a microscope but have different clinical trajectories.
E. **Integrated “Layered” Diagnosis:** The WHO 2026 format requires a “layered” reporting structure for glioblastoma to ensure all data are captured.

**A special note is added for clinicians** that highlights “molecular GBMs” (as those meeting molecular but not histological criteria) should be treated as aggressively as those with visible necrosis on a slide, as their clinical outcomes are nearly identical.

Beyond histological features, transcriptomic profiling has identified three distinct molecular subtypes—proneural, classic, and mesenchymal—each characterized by unique genetic alterations that influence patient prognosis and treatment response [29,30]. Table 1 represents the 2021 GB classification framework detailing the essential biomarkers and the refined CNS subtypes that have emerged to better account for tumor heterogeneity and provide a more personalized diagnostic layer as well as key clinical/diagnostic changes incorporated by the WHO 2026, 6th Ed. [26,27,28].

### 2.2. Prognostic Biomarkers and Emerging Molecular Targets in Glioblastoma

Beyond diagnostic criteria, several biomarkers serve as critical indicators for therapeutic efficacy and survival because of the extensive literature search on in vivo wet lab analysis and integrative bioinformatics studies. These can be categorized as follows:

- O6-methylguanine-DNA methyltransferase (MGMT) Promoter Methylation: This is a primary predictor of response to TMZ; patients with promoter hypermethylation typically experience improved outcomes due to reduced DNA-repair activity [31,32].
- Driver Mutations: Recurrent mutations in TP53, PTEN, and EGFR drive tumorigenesis by disrupting essential pathways related to cell survival, proliferation, and invasion [33,34,35].
- Emerging Transcriptional Profiles: Recent studies have identified specific gene expression patterns in TMZ-resistant tumors, such as the upregulation of growth differentiation factor (GDF15), serum amyloid (SA) A1/2, downregulation of tyrosine kinase with immunoglobulin and epidermal growth factor homology domains 1 (*TIE1*), calcium voltage-gated channel auxiliary subunit α2Δ1 (*CACNA2D1*), calpain 6 (*CAPN6*) and a disintegrin and metalloproteinase with thrombospondin motifs 6 (*ADAMTS6*), which may serve as novel therapeutic targets [36].
- Epigenetic Regulators: MicroRNAs (miRNAs) have emerged as vital post-transcriptional regulators that can function as either oncogenes or tumor suppressors, offering potential for future targeted therapies [37,38].

**Figure 2:**
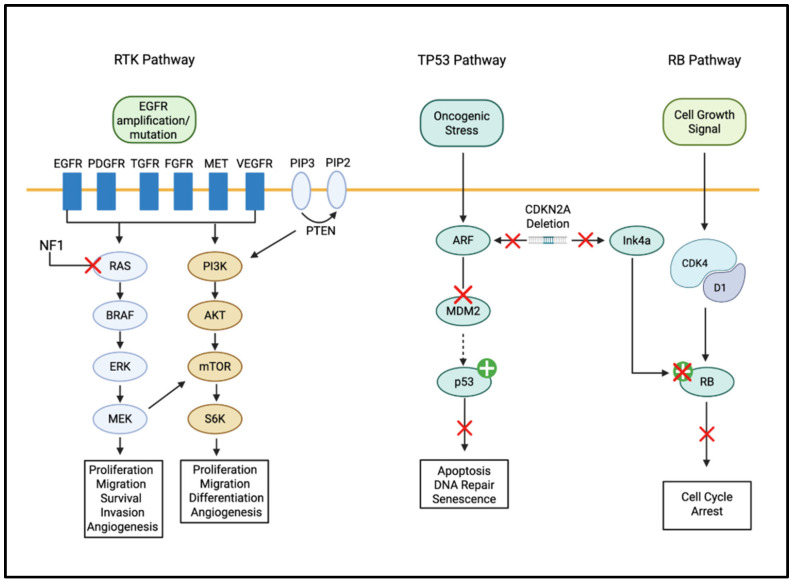
Key signaling pathways affected in glioblastoma. (Created in BioRender. Ranjan, A. (2026) https://BioRender.com/x14d09j).

Despite these advances, the rarity of certain mutations in primary GB and the high degree of intratumoral heterogeneity remain significant challenges in translating these findings into routine clinical practice. Furthermore, many genes involved in one pathway may also be implicated in other pathways, and these pathways interact and overlap to some extent. Figure 2 summarizes key signaling pathways affected by GB which could be targeted for therapeutic benefits. Furthermore, GB tumors possess unique structural characteristics, most notably a well-defined necrotic core, significant microvascular proliferation, and extensive infiltrative seeding into the surrounding brain parenchyma. This information is extensively discussed in the literature [2,17,20,31].

The diagram illustrates the core oncogenic signaling architecture driving glioblastoma (GB) pathogenesis. **(A) Upstream Activation:** At the cell membrane, Receptor Tyrosine Kinases (RTKs), most notably the Epidermal Growth Factor Receptor (EGFR), undergo amplification or mutation, triggering downstream cascades. **(B) Intracellular Cascades:** The Phosphoinositide 3-Kinase (PI3K)/Protein Kinase B (AKT)/Mammalian Target of Rapamycin (mTOR) axis is shown as a central regulator of protein synthesis and anti-apoptotic signaling. Parallel activation of the Rat Sarcoma Virus (RAS)/Mitogen-Activated Protein Kinase (MAPK) pathway drives mitogenic signaling and uncontrolled cellular proliferation. **(C) Cell Cycle Dysregulation:** The legend highlights the frequent inactivation of the Tumor Protein p53 (p53) and Retinoblastoma Protein (RB) pathways, which bypasses critical DNA damage checkpoints and apoptotic induction. **(D) Downstream Effects:** These signaling intersections converge to promote pro-angiogenic signaling (e.g., VEGF) and the recruitment of immunosuppressive elements within the tumor microenvironment. Labeled arrows indicate stimulatory phosphorylation events, while T-bars represent inhibitory interactions lost during malignant transformation. X indicates down regulation and + indicates upregulation.

### 2.3. Therapeutic Evasion: Investigating PI3K/AKT/mTOR and Beyond

GB pathogenesis is driven by the dysregulation of several interconnected signaling pathways that control cell growth, survival, and invasion. The in vitro and in vivo analyses using specific GB cell lines and omics studies show major pathways that include the following:

- RTK/RAS/PI3K Pathway: This is the most frequently altered pathway in GBM. It is often triggered by EGFR amplification or mutations, leading to the activation of PI3K and AKT. These molecules signal the cell to increase proliferation and resist programmed cell death [39,40]. p53 Signaling Pathway: Mutations in TP53 or alterations in its regulators (like mouse double minute (MDM2)) disrupt the cell’s ability to repair DNA damage or undergo apoptosis. This allows damaged cells to continue dividing, leading to rapid tumor growth [41].
- RB (Retinoblastoma) Pathway: This pathway controls the cell cycle transition. Alterations in this pathway, such as the loss of CDKN2A, prevent the “braking” mechanism of the cell cycle, leading to uncontrolled cellular replication [20,42].
- PTEN Regulation: The phosphatase and tensin homolog **(PTEN**) gene normally acts as a tumor suppressor by inhibiting the PI3K pathway. In many GB cases, PTEN is lost or mutated, which “removes the brakes” on cell survival and promotes aggressive tumor infiltration into healthy brain tissue [20,34].

While preclinical results for many clinically relevant biomarker inhibitors like PI3K/AKT/mTOR/MGMT/EGFR inhibitors in GB were promising, clinical trials using these agents alone or in combination have largely failed to improve patient outcomes. This lack of success is primarily attributed to GB’s significant genetic heterogeneity, its invasive spread into surrounding brain tissues, and various pharmacological barriers [20,34,43,44,45]. To address these challenges, current research has shifted toward multi-target trials investigating PI3K/AKT/mTOR inhibitors in both newly diagnosed and recurrent GB to better evaluate metabolic responses and biomarkers.

### 2.4. The Hippo Signaling Pathway: A Hub for Multidrug Resistance and Therapeutic Targeting

Another crucial metabolic pathway that has been extensively studied is the Hippo pathway, a critical regulator of GB progression, where its core components, yes-associated protein (YAP) and transcriptional co-activator with PDZ-binding motif (TAZ), translocate to the nucleus to drive the expression of genes associated with cell proliferation, invasion, and the maintenance of glioma stem cells. This pathway plays a central role in multidrug resistance (MDR) by upregulating anti-apoptotic proteins like myeloid cell leukemia protein 1 (MCL-1) and activating downstream targets that bypass the cytotoxic effects of standard treatments like TMZ [46]. Furthermore, the Hippo signaling network integrates various oncogenic inputs, such as EGFR and mTOR signaling, to promote a radioresistant phenotype and create an immunosuppressive microenvironment that facilitates tumor recurrence. Principle pharmacological therapies targeting the Hippo pathway include Verteporfin which crosses the BBB and accumulate in the brain, and act on YAP/TAZ, Valproic acid which reduces CD44 expression) and Amlexanox which affects actin cytoskeleton remodeling. Other agents include ALX which regulates post-translational control of large tumor suppressor Kinase 1 and 2 (LATS1/2) and inhibits nuclear factor kappa-B kinase subunit epsilon (IKBKE); Bazedoxifene(which inhibits glycoprotein 130 (GP130) to deactivate IL-6/GP130 and accelerate YAP phosphorylation; Nitidine chloride which increases YAP phosphorylation and Silibinin which induces a concentration-dependent downregulation of YAP [46,47]. While pharmacological agents like Verteporfin and Bazedoxifene have demonstrated robust inhibition of the YAP/TAZ axis in vitro, their clinical translation remains hampered by inconsistent BBB permeability. Current evidence for these agents sits at a Class II/III level, largely confined to preclinical models or early-phase trials. While the ‘biological potential’ for resensitizing tumors to TMZ is high, the ‘clinical readiness’ is moderate at best, as the therapeutic window required to inhibit Hippo signaling without systemic toxicity has yet to be clearly defined in human cohorts.

### 2.5. MicroRNAs as Post-Transcriptional Regulators and Therapeutic Targets

MicroRNAs (miRNAs) function as critical post-transcriptional regulators in GB, acting as either oncogenes or tumor suppressors to influence epigenetic landscapes and therapeutic resistance. Given their ability to target multiple gene networks simultaneously, miRNAs represent promising biomarkers and versatile therapeutic targets for modulating the complex biological processes driving GB progression [48,49]. The key miRNAs investigated for GB therapy include *miR-21* (acts by targeting tumor suppressors like PTEN and PDCD4) and *miR-10b* (essential for glioma cell viability). Research suggests GB is “addicted” to miR-10b, and its inhibition via gene editing (CRISPR-Cas9) or antisense oligonucleotides has shown lethal effects on tumor cells. Others include *miR-34a* (inhibits tumor growth and induces cell death by targeting oncogenes like c-Met and Notch), *miR-124* (acts as a tumor suppressor by inducing cell cycle arrest through the targeting of CDK4 and CDK6), *miR-7* (one of the most potent tumor suppressors in GB, it regulates multiple pathways including *PI3K/AKT* and the MAPK cascade (*RAF/MEK/ERK)* by targeting oncogenes such as EGFR and RAF-1) and *miR-128* (targets the *Bmi-1* oncogene and helps regulate cell differentiation and proliferation) [48,49,50,51,52].

miRNA-based therapeutic biomarkers represent a high-impact frontier for GB, offering a Class II evidence level for their ability to simultaneously regulate multiple oncogenic networks and provide precise diagnostic signatures through liquid biopsy. While their biological effective size is significant, clinical readiness is currently constrained by the “naked” miRNA stability problem and the need for standardized isolation protocols. Consequently, their successful translation remains highly dependent on the advanced nano-delivery systems required to ensure serum stability and efficient endosomal escape. 

## 3. Targeted Therapy Using Biomimetic Nano-Formulations (BNFs)

### 3.1. Rationale and Key Design Principles

The efficacy of current GB therapies is constrained by: (i) poor treatment specificity, leading to collateral damage of healthy brain tissue and severe side effects; (ii) the infiltrative nature of GB, which prevents complete surgical resection and drives recurrence; (iii) multidrug resistance and tumor recurrence driven by glioma stem cells (GSCs); and (iv) the restrictive BBB, as well as the blood–tumor barrier (BTB), which is created by aberrant vasculature and elevated interstitial pressure within the tumor microenvironment and limits chemotherapeutic delivery [53,54,55,56,57,58,59,60,61,62]. Biomimetic strategies offer potential solutions by improving drug stability and solubility, enabling transport across the BBB/BTB, and reducing off-target toxicity [63,64,65,66]. BNFs address these by decorating or cloaking nanoparticle (NP) cores with biological membranes or ligands to confer native surface proteins, receptors, and “self” signals that enable immune escape, receptor-mediated transcytosis across the BBB, and homologous targeting to GB cells. Common BNF cores include liposomes, polymeric NPs (Poly (lactic-co-glycolic acid (PLGA)), inorganic cores (gold, iron oxide), and hybrid constructs; cloaks are derived from cancer cells, erythrocytes, platelets, leukocytes (macrophages, neutrophils), or engineered vesicles [67,68,69].

### 3.2. Mechanisms of Action and Targeting Strategies of BNFs

- **Homotypic targeting**: tumor cell membrane coatings present adhesion molecules that preferentially bind parental tumor cells, improving accumulation in GB tissue [70].
- **Immune camouflage and prolonged circulation**: erythrocyte or platelet membranes provide “self” markers (e.g., CD47) to reduce phagocytosis and extend half-life [71].
- **BBB transcytosis**: surface display of ligands for BBB transporters (transferrin receptor, low-density lipoprotein receptor (LDLR)-related proteins) or use of cell carriers (macrophage-membrane, neutrophil-mimics) promotes receptor-mediated transcytosis or “Trojan-horse” migration into the brain [72].
- **Multimodal payloads**: BNFs are platforms for chemotherapy (TMZ, paclitaxel), siRNA/CRISPR payloads, metabolic inhibitors, photosensitizers or radionuclides, enabling combination therapy and theranostics [73].

### 3.3. Lipoprotein-Based Nanoparticles

Most reviews overlook the importance of lipoprotein-based nanoparticles, specifically reconstituted low-density lipoproteins (rLDL) and high-density lipoproteins (rHDL) and their mimetics. As a biologically inspired platform for GB therapy, these nanoparticles exploit endogenous lipid transport pathways to enhance both blood–brain barrier (BBB) penetration and tumor selectivity. These particles can incorporate hydrophobic chemotherapeutics, photosensitizers, radionuclides, or nucleic acid cargos in a lipid core and present apolipoproteins (or their mimetic peptides) on the surface to engage LDLR or scavenger receptor class B type 1 (SR-B1), which are frequently upregulated on BBB endothelium and glioma cells [74]. Receptor-mediated transcytosis and selective lipid uptake enable enhanced intratumoral delivery relative to free drug and may reduce systemic toxicity by redirecting payloads away from non-target tissues [74,75,76]. HDL-like nanoparticles are especially attractive for delivering cholesterol-conjugated small interfering RNAs (siRNAs) and lipophilic small molecules because SR-B1-mediated selective lipid transfer can bypass endo-lysosomal sequestration, enhancing cytosolic delivery [76]. LDL-mimetic carriers are well suited for highly lipophilic drugs and prodrugs that exploit LDLR-mediated endocytosis in proliferating tumor cells [77]. Key translational challenges include scalable production with reproducible drug loading, avoiding rapid hepatic clearance (native lipoprotein tropism), heterogeneity of receptor expression across GB subtypes, and achieving uniform distribution into infiltrative tumor margins. Combining lipoprotein mimetics with tumor-targeting ligands, stimuli-responsive release mechanisms, or multimodal therapies offers a rational path toward improving efficacy in preclinical GB models and advancing toward clinical evaluation [77,78,79,80,81,82].

### 3.4. Translational Status and Clinical Considerations

Selected studies demonstrate improved outcomes in orthotopic GB models: engineered BNFs have delivered synergistic metabolic inhibitors and chemotherapies to reduce tumor growth and extend survival; cell-membrane-coated liposomes have improved fluorescence-guided resection and local control; and macrophage-membrane-camouflaged NPs have modulated tumor-immune microenvironments while delivering payloads across the BBB [83,84,85]. Collectively, preclinical data show higher tumor deposition, reduced systemic toxicity, and enhanced therapeutic indices versus naked NPs [83,84,85,86,87,88]. Immunomodulation and combination with immunotherapy using BNFs has shown promise in transporting immunostimulatory agents (STING agonists, checkpoint inhibitors, tumor antigen peptides) or in presenting tumor antigens directly via cancer cell membrane coatings, thereby bridging delivered cytotoxic therapy with antigen presentation and immune activation. Early work indicates that biomimetic formulations can favorably reprogram the GB microenvironment, but overcoming GB’s profound immunosuppression remains a major challenge [71,89].

### 3.5. The Translational Gap: Biological Hurdles and Model Limitations

While multiple biomimetic strategies have shown robust efficacy in preclinical settings, clinical translation remains elusive, largely due to a significant biological “translational gap” that is often under-interrogated. Standard orthotopic GB models frequently utilizing immunocompromised mice and homogeneous cell lines fail to replicate the complex architecture of human tumors. Specifically, human GB is characterized by a “leaky” but heterogeneous BTB and high interstitial fluid pressure that differs significantly from the relatively uniform BBB disruption seen in rodent models. Furthermore, the biological reasons for the failure of nanomedicines in humans involve species-specific differences in the immune microenvironment. In humans, the “protein corona” that forms around nanoparticles upon injection is governed by a distinct proteome compared to mice, often leading to rapid sequestration by the mononuclear phagocyte system (MPS) despite biomimetic “self” signaling. Additionally, the dense extracellular matrix (ECM) and the specialized perivascular niches in human GB present physical barriers to nanoparticle penetration that are not adequately captured in short-term animal studies. Addressing these requires a shift toward human-on-a-chip models or porcine models, which better mimic the human brain’s white-to-gray-matter ratio and vascular physiology [88,89,90].

In addition, other key translational barriers include: (i) scalable reproducible methods for membrane isolation and consistent coating; (ii) long-term safety and immunogenicity of heterologous membranes; (iii) regulatory classification (biologic vs. device vs. drug); and (iv) manufacturing costs and sterility controls. A handful of nanomedicines for GB (non-biomimetic liposomal formulations, convection-enhanced devices) have entered clinical trials, but, to date, biomimetic NPs remain largely preclinical; rigorous GLP toxicology and well-designed early human studies will be essential [88,89,90].

## 4. Advances in Cancer Stem Cells (CSCs) for GB Therapeutics

### 4.1. Harnessing Stem Cell Tropism for Targeted GB Therapy

Stem-cell-based approaches for GB are rapidly evolving from conceptual preclinical strategies toward early clinical translation. Numerous studies have demonstrated that the malignant CSC subset of tumor cells in GB is likely associated with malignant recurrence and resistance to conventional treatments because of their unique capacities for self-renewal, differentiation, growth, and progression [91,92]. Two principal paradigms dominate current work: (1) stem cells as therapeutic carriers—using neural stem cells (NSCs) or mesenchymal stem/stromal cells (MSCs) to deliver oncolytic viruses, prodrug-converting enzymes, cytokines or radiopharmaceuticals directly to invasive tumor foci—and (2) modulation of GSC biology either by targeting GSC niches or by using engineered cells/vesicles to reprogram the microenvironment [93,94,95,96,97]. Preclinical and early clinical studies through 2023–2024 show reproducible tumor tropism of NSCs/MSCs and therapeutic benefit in animal models, which has driven several first-in-human safety and feasibility trials [96,97,98,99,100].

### 4.2. Technological Advances Improving Delivery and Safety of GSCs

(a) *Genetic engineering of stem cells* to express oncolytic viruses (or to carry replication-competent viral payloads) or suicide enzymes that convert systemically administered prodrugs into cytotoxins locally, thereby increasing intratumoral drug concentration while reducing systemic toxicity [101,102,103,104,105];
(b) *Locoregional administration* (intra-tumoral, resection-cavity, intraventricular) to maximize contact with infiltrative cells; and
(c) *Encapsulation/biomaterial scaffolds* to prolong cell survival and controlled release. These strategies have enhanced persistence and antitumor efficacy in multiple animal models and underpin several clinical trial designs [101,102,103,104,105,106].

### 4.3. Biomimetic Delivery via MSC Platforms: Opportunities and Safety Considerations

MSCs and MSC-derived extracellular vesicles (EVs) have attracted attention because of manufacturing scalability and immunomodulatory properties. MSCs can home to tumors and be loaded with small molecules, RNAs, or oncolytic agents; MSC-EVs offer a cell-free alternative that may reduce risks of unwanted engraftment while retaining payload delivery and BBB crossing potential. Preclinical evidence indicates that MSC/EV platforms slow tumor growth and sensitize GB to other therapies, and early-phase trials exploring allogeneic MSC products and MSC-based delivery are now registered. However, MSCs have context-dependent effects and in some settings may promote tumor-supportive inflammation, so careful engineering and thorough safety evaluation remain essential [107,108,109].

### 4.4. A Related and Fast-Moving Area Is Engineered Immune–Stem Cell Hybrids and Cell Therapies

Combining stem cell carriers with CNS homing patient-derived T cells represents a potent strategy for overcoming traditional delivery barriers. Additionally, organoid and patient-derived tumor models are refining target selection and predicting response to cell therapies, accelerating rational design and biomarker selection for trials. Although durable clinical responses remain limited to small cohorts, these combined engineering + delivery advances have produced multiple ongoing Phase I/II studies and a clear translational pathway toward more effective combinatorial regimens [110,111].

### 4.5. Deconstructing GB Heterogeneity Through High-Resolution Mapping

The traditional classification of GB into proneural, classical, and mesenchymal subtypes—while foundational—is now understood to be an oversimplification of a highly fluid biological landscape. Single-cell RNA sequencing (scRNA-seq) has revolutionized this paradigm by revealing that these “subtypes” actually coexist within a single tumor as distinct cellular states [112,113]. Research has identified four primary meta-states—progenitor-like (OPC-like, NPC-like) and differentiated-like (AC-like, MES-like)—that exhibit remarkable plasticity. These cells do not exist in fixed clones but can transition between states in response to microenvironmental cues or therapeutic pressure, explaining why therapies targeting a single genetic driver often lead to the rapid emergence of resistant subpopulations.

Spatially resolved transcriptomics has added a critical second dimension to this understanding by mapping how these cellular states are organized within the tumor architecture. This technology reveals that the GB microenvironment is a complex mosaic; for instance, mesenchymal-like cells tend to cluster in hypoxic cores or near “leaky” vasculature, where they interact with infiltrating immune cells to reinforce a local immunosuppressive niche [113]. Conversely, cells at the infiltrative margin often harbor unique genetic signatures that prioritize migration over proliferation, allowing them to evade local treatments like surgical resection and radiotherapy. By integrating these high-resolution mapping tools, it becomes clear that the “frontier” of GB therapeutics must move away from static “one-size-fits-all” molecules. Instead, this biological depth informs the development of combinatorial nano-therapeutics and multi-target delivery systems designed to address the spatial and cellular mosaicism of the tumor simultaneously.

### 4.6. Outlook and Challenges

Stem-cell-based therapies for glioblastoma (GB) are currently in the early clinical readiness stage, with several Phase I/II trials investigating neural and mesenchymal stem cells as targeted delivery vehicles for oncolytic viruses or prodrug-activating enzymes. While preclinical results are robust, the evidence quality remains low-to-moderate (Level II/III) due to a lack of large-scale randomized controlled trials, and significant limitations persist regarding the optimal administration route, potential for stem-cell-induced tumor growth, and the immunosuppressive nature of the GB microenvironment. The transition of CSC-targeted therapies from bench to bedside is hindered by fundamental differences in stem cell niche biology across species. While mouse models demonstrate successful GSC eradication, the human GB microenvironment is significantly more immunosuppressive and hypoxic, factors that protect GSCs from both cell-based carriers and molecular inhibitors [91,93]. In humans, the “infiltrative frontier” of GSCs resides in a complex neurovascular unit that is rarely recapitulated in rodent xenografts, leading to an overestimation of drug reach and persistence. Key hurdles include tumor heterogeneity, immunosuppressive microenvironment, potential for pro-tumorigenic activity of stem cells, and the documented failure of animal models to predict the human “cytokine storm” or off-target neurotoxicity. To bridge this gap, future research must move toward critically interrogating therapeutic efficacy in patient-derived organoid systems that preserve the spatial architecture of the TME. The most promising near-term strategy involves leveraging engineered stem cell carriers in tandem with agents designed to disrupt the human-specific immune shielding of the GSC niche. Continued careful early-phase trials and standardized safety reporting will determine whether stem-cell-based platforms can move beyond niche applications to meaningful survival benefit in GB [91]. 

## 5. Advances in Immunotherapy for GB

Over the past two decades, immunotherapy has revolutionized research and clinical care across various malignancies. The early stages of immunotherapy research generated palpable excitement, which was later solidified by the awarding of the 2018 Nobel Prize in Physiology or Medicine to James Allison and Tasuku Honjo [114]. Their landmark work characterized two critical proteins for immune-based therapy: Cytotoxic T-Lymphocyte-Associated Protein 4 (CTLA-4) and Programmed Cell Death Protein 1 (PD-1), respectively [115,116,117,118,119]. While initial results for hematologic malignancies and certain solid tumors, such as melanoma, were promising [120,121,122], this success has not yet been replicated in many other solid cancers [123]. Unlike many “hot” tumors characterized by immune infiltration, GB is considered an immunologically “cold” cancer, defined by limited immune activity and a profoundly immunosuppressive microenvironment [124,125,126]. Mechanistically, this ‘cold’ status is driven by several convergent factors: (i) the physical sequestration of the CNS behind a BBB that restricts lymphocyte trafficking; (ii) a lack of professional antigen-presenting cells (APCs) in the parenchyma; and (iii) the high prevalence of bone-marrow-derived macrophages (BMDMs) and myeloid-derived suppressor cells (MDSCs) that secrete TGFß and IL-10, effectively quenching T cell activation [127,128].

Early clinical trials of GB immunotherapies have largely failed to improve overall survival, due in part to the dominance of immunosuppressive macrophages, sparse T cell infiltration, and profound tumor heterogeneity [129,130,131]. Furthermore, activating immune responses within the CNS carries significant risks—including cerebral edema and neuro-autoimmunity—underscoring the precarious balance between therapeutic efficacy and patient safety [129]. Despite these setbacks, the poor prognosis of GB necessitates continued exploration. Current research focuses on diverse strategies such as chimeric antigen receptor (CAR) T cell therapies, immune checkpoint inhibitors, and peptide vaccines. As no single modality is likely to succeed in isolation, future breakthroughs will likely depend on integrating multiple immunotherapies with standard-of-care treatments, including surgical resection, chemotherapy, and radiotherapy [130,131,132].

### 5.1. Immune Checkpoint Inhibitors (ICIs)

In healthy immunological systems, immune checkpoints play a vital role in modulating immune responses, preventing them from tipping toward autoimmunity. For instance, the activity of cytotoxic T cells and Natural Killer (NK) cells is tightly regulated by well-timed checkpoints to prevent collateral cytolytic damage and inflammation once a pathological state has been cleared. However, cancers often exploit these checkpoints to suppress the anticancer functions of T cells and NK cells [132,133,134]. CTLA-4 and PD-1 are two primary checkpoint pathways that tumors utilize to evade immune surveillance. Each pathway serves to dampen T cell responses—when bound to B7 and PD-L1, respectively—thereby inhibiting antitumor activity [135]. Consequently, treatment with monoclonal antibodies designed to inhibit these checkpoints can prevent or reverse this immunosuppression. Numerous clinical trials have now evaluated the efficacy of targeting the CTLA-4/B7 and PD-1/PD-L1 axes [134,135].

The optimism surrounding ICIs has been tempered by several landmark Phase III failures. The CheckMate 498 trial, which evaluated nivolumab (anti-PD-1) versus temozolomide in patients with MGMT-unmethylated GB, failed to meet its primary endpoint of overall survival (OS). Similarly, CheckMate 548, which added nivolumab to standard of care in MGMT-methylated patients, also yielded disappointing results. These failures highlight that PD-1 blockade alone is insufficient to overcome the ‘T-cell exhaustion’ and low tumor mutational burden (TMB) characteristic of GB. Future ICI application likely requires identifying specific ‘hypermutator’ phenotypes or using neoadjuvant dosing to ‘prime’ the immune system prior to resection [136].

To date, immune checkpoint blockade has shown limited efficacy as a standalone therapy for GB. No studies have conclusively demonstrated that CTLA-4 or PD-1/PD-L1 monoclonal antibodies significantly improve overall survival in this patient population. Nonetheless, these early trials have advanced our understanding of both GB immunology and the challenges of antibody-based therapies. Current efforts are focused on combining checkpoint inhibitors with other immunotherapies and standard treatments, while emerging targets such as TIGIT and LAG3 are under active investigation [137,138,139]. Our search for novel GB targets identified cell-surface-proliferating cell nuclear antigen (csPCNA) as a promising target for future immunotherapy using NK cells [140].

### 5.2. Chimeric Antigen Receptors (CAR)

These are genetically engineered receptors that allow for the artificial selectivity of T cell and NK cell activation and proliferation against tumor cells, often specific subtypes of antigen-expressing cancers. The CAR receptors contain a designer construct with linked segments: an extracellular ligand-binding domain (often a single-chain variable fragment of an immunoglobulin, which gives the receptor its high degree of specificity for its target), a transmembrane portion, and an intracellular portion that interacts with the activation signaling cascades of T cells and NK cells. The specifics of the molecular construct contained within the CAR has evolved from the first generation to the fourth and fifth generations, which now contain the molecular machinery to both activate the CAR-expressing cell and stimulate the cell to proliferate (via the inclusion of tools to push cytokine pathways) [141,142]. Most previous studies have focused on using T cells as the cell of interest, but, in recent years, NK cells have also been studied for their ability to be a more “off-the-shelf” therapy since they are not restricted to use in a single patient (like T cells are due to HLA restriction) [143,144]. Like immune checkpoint inhibitors, CAR-T cell therapies have demonstrated success in blood cancers but, overall, have so far demonstrated narrow success in solid tumors, including GB [145,146]. Clinical trials have been performed and many are still ongoing. The main targets of interest have been EGFRvIII, HER2, IL-13R⍺2, EphA2, and GD2 [147,148,149]. A recent work from the Massachusetts General Hospital, which used CAR T cells designed to target EGFR and EGFRvIII via a T-cell-engaging antibody molecule (termed ‘CARv3-TEAM-E T cells’) demonstrated a “dramatic and rapid” regression of the GB tumor [150]. Moving forward, GB-directed CAR research is centered on three priorities: identifying novel and truly tumor-specific antigens, engineering CARs resilient to the hostile GB microenvironment, and combining CARs with other immunotherapies or standard-of-care approaches such as surgery, radiation, and chemotherapy. Collectively, these efforts reflect a recognition that overcoming the biological barriers of GB will require highly engineered multi-pronged CAR strategies rather than single-agent approaches.

### 5.3. Tumor Vaccines

Cancer vaccines aim to trigger an immune response against tumor antigens, functioning much like infectious disease vaccines but targeting tumor-specific (TSAs) or tumor-associated antigens (TAAs). In GB, vaccines introduce these antigens to antigen-presenting cells, primarily dendritic cells (DCs), which then activate CD8+ and CD4+ T cells to mount a coordinated antitumor response. This strategy is particularly relevant for GB, where immune evasion and a highly suppressive microenvironment limit spontaneous T cell activity [151,152,153].

GB vaccine approaches are commonly classified by antigen type—such as tumor-specific antigens (TSA), tumor-associated antigens (TAA), or pathogen-derived antigens—and platform type, including peptides, dendritic cells (DCs), nucleic acids, or viral vectors [153]. Peptide vaccines deliver tumor antigens alongside adjuvants to enhance T cell activation; notable GB candidates include Rindopepimut (targeting EGFRvIII), SurVaxM (targeting survivin), and HSPPC-96. DC vaccines, such as DCVax-L (loaded with autologous tumor lysates), have shown encouraging survival benefits in Phase III trials. Nucleic acid vaccines, primarily mRNA-based, encode tumor antigens and are currently being evaluated in over a dozen GB clinical trials [154,155,156,157,158,159,160,161,162]. Furthermore, viral vector vaccines employ engineered viruses to introduce tumor antigens, with a cytomegalovirus-based platform currently under investigation. Together, these strategies highlight the diversity of vaccine platforms being developed to overcome the immune resistance inherent to GB. Despite the theoretical appeal of targeted vaccines, the ACT IV Phase III trial of Rindopepimut (targeting the EGFRvIII mutation) was terminated early due to a lack of survival benefit compared to the control group. This failure is largely attributed to ‘antigenic drift’ or ‘immune escape,’ where the tumor selectively outgrows EGFRvIII-negative clones once the targeted population is eliminated. This underscores the limitation of single-antigen targeting in a tumor as heterogeneous as GB [161].

### 5.4. Oncolytic Virotherapy (OV)

Oncolytic virotherapy is gaining momentum as a novel treatment strategy for glioblastoma (GB), offering both direct tumor lysis and immune activation. A landmark in the field was the approval of the herpes-simplex-virus-derived G47Δ (Delytact/Teserpatreuv) in Japan for malignant glioma, providing proof of concept that intratumoral viral therapy can yield clinical benefit [163]. Other candidates such as PVSRIPO, a recombinant poliovirus chimera, and DNX-2401 (Delta-24-RGD), an adenovirus vector, have shown evidence of durable responses and are being tested in combination with checkpoint inhibitors such as pembrolizumab to enhance efficacy [164].

Recent advances include next-generation engineering of OVs armed with cytokines, immune stimulatory molecules, or tumor-selective promoters to heighten immunogenicity and persistence in the hostile GB microenvironment. Modifications to viral capsids and tropism are also being used to improve tumor targeting and evade neutralizing immunity. Early results suggest that these approaches can convert GB from an immunologically “cold” to a more “inflamed” tumor, improving T cell recruitment and response durability [165,166,167,168]. Despite challenges—including delivery barriers, pre-existing antiviral immunity, and GB heterogeneity—oncolytic viruses represent a rapidly advancing area. Future directions emphasize rational combinations with checkpoint inhibitors, vaccines, cell therapies, and standard-of-care modalities. Collectively, these strategies position Ovs as an emerging practical component of multimodal GB therapy.

Overall, immunotherapy in GB currently functions as a high-potential frontier with a Class I/II evidence level, transitioning from failed monotherapies to promising personalized vaccines and combinatorial “neoadjuvant” approaches. Its clinical readiness remains hindered by the “cold” immunosuppressive microenvironment and antigenic heterogeneity, necessitating synergistic strategies like blood–brain barrier disruption to achieve meaningful therapeutic efficacy.

## 6. Bioinformatics and Machine Learning in GB Therapy

Bioinformatics and machine learning (ML) have emerged as powerful tools to unravel GB complexity and guide therapeutic innovation. While these approaches are being used in glioblastoma, the central theme of many approaches is to use retrospective data for prospective clinical decision making. A high percentage of the recent literature centers around patient survival prediction because this is an extremely important aspect for a cancer as challenging as GB [169,170]. Accurate information about survival, leveraged from large populations, can allow the patient and provider to plan. Accurate prognostication also allows the patients and their loved ones to prioritize what they deem most important and determine what they value during the affliction with this deadly tumor.

### 6.1. High-Throughput Omics Technologies

Genomics, transcriptomics, epigenomics, proteomics, and metabolomics—generate massive datasets that can capture the molecular landscape of GB [171]. Bioinformatics pipelines enable the integration and analysis of these datasets, identifying key drivers such as EGFR, PTEN, TP53, and IDH mutations, as well as epigenetic signatures (e.g., MGMT promoter methylation). Beyond single-gene markers, systems-level analyses uncover dysregulated pathways (e.g., PI3K/AKT/mTOR, WNT, DNA repair networks) that are crucial to therapeutic targets. Multi-omics integration enhances patient stratification, enabling the discovery of subtypes with distinct prognostic and therapeutic responses [172,173,174].

### 6.2. Machine Learning (ML) in GB Survival Prediction: Performance vs. Generalizability

ML enables the integration of demographic, clinical, and molecular features to improve survival prediction in GB [175,176]. In a cohort of 135 patients, XGBoost achieved the highest performance measured as receiver operating characteristic (ROC) and area under the curve (AUC) (ROC-AUC 0.90, accuracy 0.78) when combining age, Karnofsky Performance Status, and markers such as MGMT methylation and EGFR amplification. While the approach shows promise, its generalizability is limited by reliance on a single-institution dataset without external validation [169]. However, such high-performance metrics in small single-institution cohorts often reflect overfitting to local data rather than true predictive power. Directly comparing these ROC-AUC values across studies is inherently misleading due to fundamental differences in patient demographics, varying clinical endpoints (e.g., progression-free vs. overall survival), and the lack of standardized preprocessing, which prevents these metrics from serving as universal benchmarks. These values cannot be directly compared to other studies due to significant variations in patient populations and clinical endpoints. Li et al. addressed the challenge of external validation by training survival prediction models on 183 GB patients and validating them on independent cases from The Cancer Imaging Archive (TCIA), achieving AUC values above 0.80, though accuracy decreased when clinical risk factors were included, likely due to limited sample size [177]. Expanding on this, the ReSPOND consortium analyzed 2838 patients from 22 institutions, applying standardized MRI processing and radiomic feature extraction, including novel metrics such as ventricular proximity, to stratify patients into prognostic subgroups. This approach yielded a concordance index of 0.64 with imaging features alone, which improved to 0.67 when molecular, demographic, and surgical data were incorporated. The disparity between the high AUCs seen in small-scale studies and the lower indices in large-scale consortia underscores the ‘translational gap’ in AI; models often fail to maintain performance when faced with the real-world heterogeneity of multi-institutional data. Nonetheless, incomplete molecular profiling and omission of treatment-adaptive variables limit model generalizability, emphasizing the need for multimodal multi-institutional approaches. Furthermore, the clinical adoption of these tools faces significant regulatory hurdles, as most current models lack the prospective validation and explainability required for high-stakes oncological decision making.

### 6.3. Computational Frontiers in GB: Leveraging AI for Precision Oncology

ML algorithms extend these insights by leveraging high-dimensional datasets to build predictive models. Supervised learning methods (e.g., random forests, support vector machines, deep neural networks) can classify tumors, predict survival, and estimate therapy response based on molecular, imaging, and clinical data. Unsupervised learning (e.g., clustering, autoencoders) has been used to identify novel GB subtypes and therapeutic vulnerabilities. Deep learning applied to radiomics and pathology images further enables the non-invasive prediction of genetic alterations and treatment outcomes. Importantly, ML-based drug repurposing pipelines have identified candidate compounds by integrating omics, drug–target interactions, and gene expression perturbations [178,179]. Clinical translation is being advanced by ML-driven adaptive trial designs and biomarker-guided therapy selection. For example, patient-derived xenografts and organoid models can be paired with ML-based drug sensitivity predictions to personalize treatment. Moreover, network-based bioinformatics approaches support rational drug combinations to overcome resistance.

In a well-articulated study, Castro et al. used genomic and computational modeling of 274 patients with MGMT-methylated GB to predict responses to temozolomide, lomustine, and their combination. Biosimulation revealed wide variability, with some patients showing strong synergy and others negligible or even harmful effects from combination therapy. The findings suggested that AI-driven biosimulation could help tailor chemotherapy, avoiding overtreatment while identifying patients most likely to benefit from combination regimens [180]. Another recently published study compared outcomes between molecular glioblastomas (molGB) and classic histologic glioblastomas (histGB) in 132 patients treated with standard chemoradiation. Overall survival was similar between groups, but molGB without contrast enhancement on MRI showed notably longer survival. Importantly, AI models reliably distinguished non-enhancing molGB from low-grade gliomas, highlighting their potential to improve diagnostic accuracy and guide clinical management [181]. In summary, machine learning approaches are continually being applied to the complex clinical treatment of GB. The potential opportunities to enhance prognostication, personalize treatment, and integrate various multimodal data sources underscore the utility of ML in this realm. While the potential is great, many challenges do remain. In addition to safely sharing available data and standardizing pipelines, limited external validation, incomplete datasets, and data generation across institutions do appear to hamper progress. However, many of these limitations can be alleviated through multi-institutional collaborations, the sharing of data, and the integration of multiple data types. These approaches can be beneficial in translating these models from research tools into highly reliable clinical aids. Currently, the translational spectrum for machine learning (ML) and bioinformatics in neuro-oncology is defined by a high effective size in data integration but a variable level of clinical readiness depending on the specific application. High-quality Class I/II evidence exists for ML in radiomics and automated segmentation, where AI-driven tools for tumor volume measurement are already nearing clinical standard of care due to their high reproducibility. In contrast, bioinformatics and multi-omics pipelines for risk stratification (e.g., predicting the Hippo-pathway-driven resistance discussed earlier) that currently provide deep biological insights but only meet Class II/III evidence level. They still lack the prospective validation required for independent clinical use. The primary translational hurdle is the “black box” nature of complex algorithms, which often complicates regulatory approval (e.g., FDA 510 (k) clearance) that requires rigorous prospective validation and demonstrated safety in diverse real-world settings. Furthermore, clinical deployment is stalled by the lack of seamless integration into Electronic Health Record (EHR) workflows, unresolved questions of legal liability for AI-driven errors, and the high computational cost of maintaining these tools in resource-limited hospital environments. While the effective size of ML in identifying novel biomarkers like miRNA signatures is significant, true clinical translation is currently hampered by the lack of standardized “clean” datasets that represent the full spatial and temporal heterogeneity of glioblastoma [182].

## 7. Radiotherapeutics Advances in GB

Radiotherapy (RT) has been a cornerstone in the treatment of glioblastoma for many years [183]. However, it has now been seen that conventional radiation therapies often yield below-optimal results in treating these patients and have debilitating side effects due to off-target radiation. Over the past several decades, significant advances in RT treatment and image guidance technology have led to enormous improvements in early clinical use to improve local tumor control, enhance treatment precision, and reduce toxicity [184]. In this section, we highlight these areas and the future developments in radiation therapy for GB.

### 7.1. Proton and Heavy Ion Therapy:

Proton therapy (PT) and heavy ion therapy are promising emerging methods that offer precise dose delivery with minimal exit dose to reduce off-target irradiation, potentially saving healthy brain tissue from unnecessary radiation [184,185]. Within the last decade, another RT has emerged, known as the FLASH RT, to decrease damage to normal tissue while maintaining similar tumor control, resulting in a widened therapeutic index. This radiation therapy uses ultra-high dose rates (~40–60 Gy/s), which demonstrates remarkable normal tissue sparing in preclinical studies while maintaining similar tumor control compared to dose-matched conventional rates (~2 Gy/min) [186,187,188,189]. Similarly, carbon ion radiotherapy (CIRT) has been reported to have higher biological effectiveness, and many researchers claim that it may be able to overcome GB radio resistance [190]. The investigators hypothesized that the induction CRIT boost would result in a greater initial tumor destruction ability and prime the tumor microenvironment for enhanced immunologic tumor clearance, resulting in an expected 33% improvement in the overall survival (OS) rates. Another study by Guo et al. investigated the effects of X-ray and CIRT on the glioblastoma cell line U251 to evaluate the distinctive advantages of carbon ion treatment and explore mechanisms for overcoming radiation resistance [191].

### 7.2. Cold Atmospheric Plasma (CAP)

CAP is an emerging oncological tool that utilizes ionized gas to deliver reactive oxygen and nitrogen species (RONS), selectively inducing apoptosis in GB cells while sparing healthy tissue [192]. The technology is currently in the pre-clinical-to-early-clinical stage, with most data derived from in vitro and mouse xenograft models, though it is transitioning toward first-in-human pilot studies [193]. Regarding effective size, CAP is primarily limited by its shallow penetration depth of only from three to five cell layers, making it best suited as an intraoperative adjuvant for treating the “surgical bed” rather than bulk tumor masses [194,195]. Consequently, its current evidence level is generally considered Level IV or V (expert opinion and laboratory/animal studies), as robust Phase II/III clinical trial data are still pending.

### 7.3. Tumor-Treating Fields + Radiotherapy

Tumor-Treating Fields (TTFields) is an approach, approved by FDA, in which alternating electrical fields exert biophysical force on charge and polarizable molecules known as dipoles, which has been demonstrated to extend survival for patients with newly diagnosed GB, recurrent GB and mesothelioma [196]. The ability of TTFields to strengthen antitumor immunity, increase the permeability of the cell membrane and the BBB, and disrupt DNA repair mechanism makes it a promising therapy when combined with conventional treatment [197]. Presently, TTFields, being noninvasive and innovative, has emerged as the fourth most effective treatment option for the management of GBs. However, the overall acceptance of TTFields in real-world clinical practice is still low [198,199].

While TTFields are recognized in major clinical guidelines (like National Comprehensive Cancer Network (NCCN)), their real-world implementation is fraught with skepticism. The controversies generally center on the disconnect between the “idealized” results of the EF-14 clinical trial and the practical realities of long-term patient commitment, economic strain, and uneven global adoption [195]. (a) Adherence burden: To achieve significant survival benefits, patients are advised to wear the device for at least 18 h a day (an adherence rate of 75%). Research from academic centers in 2024 and 2025 indicates that many real-world patients fall below this threshold [199,200]. Beyond the physical weight of the battery pack, patients face “invisible” burdens like frequent hair shaving (every 2–3 days), skin irritation (dermatitis in up to 50% of users), and the social stigma of a visible head-mounted device. Additionally, technological failures in real-world usage are also introduced by external factors like technical errors during hot weather, which can lead to device overheating and forced downtime. (b) Cost-effectiveness: TTFields is one of the most expensive adjuvant therapies in oncology, with monthly costs ranging from $21,000 to $26,000. This has created a massive debate over its “value” versus “cost.” (c) Persistently low clinical uptake: the expert gap in the in the neuro-oncology community highlights that, while the EF-14 trial was positive, many experts found the interpretation of the results “provocative” or biased due to the lack of a true “sham” (placebo) control in the clinical trials [200]. There are concerns that trial participants represent a “super-compliant” subset of the population that does not reflect the average, elderly, or frail GB patient seen in daily practice (generalizability issues).

### 7.4. Boron Neutron Capture Therapy (BNCT)

BNCT is a biochemically targeted radiotherapy that utilizes the nuclear reaction between administered boron-10 and external thermal neutrons to selectively destroy glioblastoma (GB) cells at the cellular level [201,202]. Its clinical readiness has recently shifted from experimental reactor-based settings to hospital-based accelerator systems, with multiple Phase I/II trials currently active in Japan, Europe, and the U.S. in early 2026 [203]. The effect size in recurrent GB is significant, with studies reporting objective response rates (ORRs) often exceeding 70% and median survival times that frequently surpass historical standard-of-care benchmarks. The current evidence level remains at Level II (well-designed Phase II clinical trials and systematic reviews) as the field still lacks definitive large-scale Phase III randomized controlled trials to establish it as a first-line standard. Despite this, its ability to target infiltrative and radioresistant cells makes it a highly promising adjunct for managing refractory brain tumors [204].

### 7.5. Radioimmunotherapy (RIT)

Radiotherapy can induce systemic immune responses, providing a strong rationale for its combination with immunotherapy. Specifically, radiotherapy can elicit potent antitumor immune activity by influencing nearly every stage of the tumor immune cycle. These effects primarily involve promoting the release and presentation of tumor antigens, enhancing immune cell activation, increasing the density of tumor-infiltrating lymphocytes (TILs), and improving T cell recognition of malignant cells [205]. Common immunotherapeutic agents currently evaluated in combination with radiotherapy for GB include PD-L1 inhibitors (durvalumab, atezolizumab, and avelumab), PD-1 inhibitors (pembrolizumab and nivolumab), and the CTLA-4 inhibitor ipilimumab [206]. Although preclinical studies have demonstrated the efficacy of these combinations, clinical evidence remains limited, with current studies showing only modest efficacy in small patient cohorts.

## 8. Advances in Imaging and Diagnostic Techniques in Neuro-Oncology

Magnetic resonance imaging (MRI) is a crucial component of contemporary diagnosis and post-treatment monitoring of GB. Even though MRI is critical to diagnose GB and monitor post treatment, its use has not been standardized, and the use of advanced neuroimaging protocols demonstrate promise for improving the longitudinal monitoring of GB following treatment [207]. The roadblock for the use of MRI may be attributed to suboptimal imaging approaches and the variability of GB formation, making it difficult to generalize findings of small sample sizes [208]. However, advanced imaging techniques such as diffusion tensor imaging (DTI) [209] and functional magnetic resonance imaging (fMRI) [210] can provide new information in our quest to identify insightful biomarkers that can lead to improved prognosis and treatment outcomes.

### 8.1. Radiomics and Radiogenomics:

Radiogenomics is an emerging translational field of research aiming to study the correlation between radiographic signature and underlying gene expression [211]. Radiomics is a discipline that aims to make intelligent predictions and derive medical insights based on quantitative features extracted from medical images to improve clinical diagnosis or outcome [212,213,214]. Radiomics and radiogenomics present a potential to precisely diagnose and predict survival and treatment responses via morphological, textural, and functional features derived from MRI data, as well as genomic data [215,216]. It can help us understand the genomic basis of gliomas, such as tumor spatial heterogeneity, treatment response, molecular classifications and tumor microenvironment immune infiltration, which, in turn, can provide crucial insight to providing best treatment options [217,218]. In today’s world, machine learning or artificial intelligence can play a substantial role in supplementing radiomics and radiogenomics in elucidating a personalized RT treatment tailored to patients [219].

### 8.2. Enhanced Neuroimaging: Indicators of BBB Integrity and Treatment Response

Conventional MRI techniques rely on gadolinium (Gd) contrast-enhanced T1-weighted (Gd-T1w) images, which show that a less than 5% signal enhancement can serve as a good marker of treatment outcomes [220]. Gd under typical conditions does not cross the BBB, rendering its use mainly to later stages of the disease [221]. The 5% benchmark most likely indicates that patients with improved outcomes as evidenced by reduced signal enhancement, also possess preserved BBB function and early markers of a positive treatment response. An insight from these references is that technologies and approaches that enable earlier detection and treatment of GB are critical. 

### 8.3. Diffusion Tensor Imaging (DTI) and Neurite Imaging: Mapping Microstructural Heterogeneity

DTI can be an important tool for the novel diagnosis and monitoring of GB. DTI is a specialized diffusion-weighted MR imaging technique, sensitizing images to the thermal movement of water molecules and allowing us to estimate a mathematical object called the diffusion tensor that describes the bulk characteristics of water translation on each voxel [222]. The translation is primarily modulated by the brain’s microstructure (cytoarchitecture), allowing us to infer cellular characteristics from the apparent translation of water molecules and MR signal changes in response to probing among distinct spatial orientations. DTI has been applied to GB and found that it can provide putative markers of metastasis [223] and tumor migration following surgery [224]. However, DTI suffers from its simplicity to provide an accurate description of the heterogenous biological underpinning of GB. To overcome the limitations of DTI, Neurite Orientation Dispersion and Density Imaging (NODDI) has been suggested [224]. NODDI estimates the brain’s microstructural features by modeling the organization of neurites and axons at each voxel. To accomplish the model, researchers estimate three distinct compartments (compared to one of DTI) that increase the interpretability of the results. NODDI provides different metrics associated with each compartment on the model. The combined parameters have shown promise as a preoperative marker of GB [225]. DTI and NODDI can be excellent advanced markers of GB that can easily be used in preclinical and clinical studies to investigate novel therapeutics for GB, but these techniques rely on the relationship between cellular and anatomical morphology and water translation, so incorporating next-generation contrast agents with DTI/NODDI can provide significant progress towards new neuroimaging biomarkers.

### 8.4. Next-Generation Contrast Agents: Enhancing Signal Augmentation in GB

Nanoparticle formulations that can be leveraged in MRI studies can improve the detection of GB tissue and subsequent monitoring post treatment. Magnetic NP are potential new approaches to a theragnostic platform for GB [226]. Magnetic NP has been shown to be promising in breast cancer [227] and has been suggested for GBM [228]. However, one limitation it is delivery mechanisms and magnetic properties, which rely on signal reduction rather than signal augmentation and can be complemented with biomimetic NP [229]. These formulations provide the benefit of improved delivery as they mimic endogenous properties of active transport and BBB infiltration capacity. Some NPs that mimic HDL can, in theory, be loaded with traditional contrast agents to improve delivery and signal-specific augmentation to develop better strategies to diagnose and treat GB [83]. In summary, MRI has been shown to be an effective complement for GB research but improved modeling in combination with novel nanocarriers can provide the significant platform needed to yield new insight into combating GB.

### 8.5. Liquid Biopsy Frontiers

This diagnostic platform in GBM represents a paradigm shift from invasive tissue sampling to the “real-time” monitoring of the tumor’s molecular landscape through the analysis of biofluids. While standard for systemic cancers, its application in GB is unique due to the restrictive nature of the blood–brain barrier; yet, it is rapidly becoming an essential tool for longitudinal patient management. Its clinical utility in GBM is mainly seen as follows. (a) *Surveillance* and *Pseudoprogression*: One of the most critical applications is distinguishing between pseudoprogression of treatment-induced inflammatory response and true tumor recurrence. Liquid biopsies can detect rising levels of tumor-specific mutations (like *TERT* or *EGFRvIII*), which signal growth long before radiological changes are definitive. (b) *Molecular Profiling*: Since GB is highly heterogeneous, a single tissue biopsy may not capture the full genetic landscape. Liquid biopsy provides a “global” snapshot of the tumor’s evolving genome, allowing clinicians to track clonal evolution and identify new therapeutic targets in real time [22,23].

The evidence level of liquid biopsy is currently at Class II/III. While the concept is proven, large-scale prospective multicenter trials are still ongoing to standardize the protocols needed for formal inclusion in clinical guidelines. The clinical readiness of liquid biopsy is high for specialized academic centers. Liquid biopsy in GB utilizes circulating DNA (ctDNA) and cell-free DNA methylation (cfDM) to capture the tumor’s genetic and epigenetic landscape, offering a high-sensitivity method for molecular classification and real-time monitoring of resistance [230,231]. Complementing these, EVs serve as robust proteomic and transcriptomic “shuttles” that readily cross the blood–brain barrier, providing a comprehensive snapshot of the tumor microenvironment. Together, these markers facilitate a non-invasive, longitudinal alternative to traditional biopsies, although their clinical readiness currently depends on overcoming low systemic concentrations and standardizing isolation protocols. Platforms for detecting circulating tumor DNA (ctDNA), cell free methylation and extracellular vesicles (EVs) are already being used in clinical trials to monitor treatment response, particularly for immunotherapy and targeted agents. This method for diagnosis in GB is limited due to the BBB hurdle [23,24]. This is because the BBB significantly limits the “shedding” of tumor material into the peripheral blood, resulting in a much lower effective size of biomarkers in GB as compared to lung or breast cancers in early stages. This may cause sensitivity issues and lead to false negative results. There is currently a lack of universal “gold standard” protocols for the isolation and analysis of GB-derived vesicles. While less expensive than a craniotomy, the high-depth sequencing required to find rare GB mutations in the blood remains costly for routine use.

## 9. Local Surgical Interventions and Cytoreductive Advancements

The current WHO classification underscores that, while molecular profiling defines the biological identity of a tumor, surgical intervention remains the primary factor influencing the efficacy of all subsequent adjuvant therapies. While current evidence links maximal surgical resection to improved life expectancy in both low- and high-grade glioma patients, specific data regarding how the extent of resection (EOR) directly impacts survival remains limited. Beyond its diagnostic utility, surgery is also critical for treating metastatic brain lesions. Modern neuro-oncological surgery has transitioned from the traditional goal of Gross Total Resection (GTR) toward supratotal resection, which aims to resect the surrounding T2/FLAIR abnormality to address the infiltrative margin of the disease. This precision is supported by advanced intraoperative technologies, including 5-aminolevulinic acid (5-ALA) (fluorescence-guided surgery) for the real-time visualization of tumor margins and the integration of intraoperative MRI and ultrasound to account for brain shift [232,233]. Ultimately, the extent of resection (EOR) serves as the strongest independent predictor of patient response to the Stupp protocol and emerging targeted treatments; without optimal cytoreduction, the clinical impact of even the most sophisticated molecularly targeted agents is significantly diminished. Beyond physical removal, the emergence of laser interstitial thermal therapy (LITT) provides a minimally invasive surgical option for deep-seated or recurrent lesions previously deemed inoperable [234]. These advancements ensure that the cytoreductive phase of treatment creates a more favorable environment for the molecular and nano-therapeutics discussed in subsequent sections.

### 9.1. Fluorescence-Guided Resection

The implementation of fluorescence-guided surgery significantly improves the extent of resection (EOR) in patients with high-grade gliomas. Research, including a systematic review by Gandhi et al., indicates that using 5-ALA as a surgical guide achieves a Gross Total Resection (GTR) rate of approximately 76.8%, consistently outperforming traditional surgical techniques [232]. Beyond physical tumor removal, 5-ALA has been linked to tangible clinical gains, extending overall survival (OS) by three months and progression-free survival (PFS) by one month. Statistical analysis by Golub et al. further confirms this superiority, showing that 5-ALA is nearly three times more effective than standard neuronavigation alone (OR 2.866). Additionally, 5-ALA is highly effective in complex cases; when used for tumors in eloquent brain structures, it raised the EOR from 57.6% to 71.2% compared to using intraoperative MRI (iMRI) alone. Ultimately, integrating these intraoperative adjuncts is a primary strategy for maximizing resection and improving patient longevity [233].

### 9.2. Stereotactic Radiosurgery (SRS) and Hypofractionation

Radiation-based, SRS and hypofractionated protocols are integral local–regional strategies that complement surgical cytoreduction. Stereotactic radiosurgery (SRS) provides noticeable tissue sparing outside of the treatment target. Hypofractionated SRS is also a widely used approach, where ablative doses of radiation are directed to the target, thereby minimizing the risk of toxicity [191,234,235,236]. A study by Jablonska, et al. evaluated the effectiveness of hypofractionated radiation therapy with concurrent temozolomide in terms of feasibility and disease control in primary glioblastoma patients with poor prognostic factors other than advanced age. Their results demonstrated that patients with poor clinical factors, other than advanced age, could be selected for hypofractionated radiotherapy. The overall survival and progression-free survival rates were similar to those in patients treated with standard fractionation, assuring good treatment adherence, low rates of toxicity and probable improved cost-effectiveness [237]. Minniti G, et al. studied GB in the elderly population as most patients with GB are older than 60 years. They demonstrated that both temozolomide and hypofractionated radiotherapy should be considered as standard treatment options in elderly patients with GB [238].

### 9.3. Laser Interstitial Thermal Therapy (LITT)

(LITT is a MR-guided minimally invasive surgical technique used to ablate glioblastoma tissue through thermal energy, particularly when tumors are located in deep or eloquent brain regions [239,240,241]. The technology is currently in a state of high clinical readiness, with platforms like NeuroBlate and Visualase already FDA-cleared and widely integrated into neuro-oncological practice. While the evidence level primarily consists of Class II and III data from prospective cohorts and retrospective series, recent studies are exploring hybrid procedures that combine LITT with open resection or immunotherapy to improve outcomes [242,243]. Regarding effective size, LITT is generally most successful for well-circumscribed lesions smaller than 3 cm in diameter, as larger or highly irregular volumes may lead to incomplete ablation or increased edema. This “cytoreductive” approach is often reserved for recurrent GB where traditional surgery is too risky, though emerging single-cell analysis suggests it may also help modulate the tumor microenvironment to enhance future treatments [243]. Overall, while not a standalone cure, LITT serves as a critical tool in the multidisciplinary management of complex high-grade GBs.

## 10. Other Emerging/Investigational Adjuvant Therapeutic Approaches

Although we have discussed various therapeutic approaches for GB in the current literature, there are various emerging adjuvant strategies for GB in preclinical or early clinical investigation stages. These have great potential as adjuvant therapy for GB in combination with standard GB treatment regimens as well with immunotherapy, nanoparticles, or oncolytic viruses to improve treatment response and survival. Some of these approaches are briefly discussed here.

### 10.1. The Ketogenic Diet (KD)

KD has been explored as an adjunctive treatment for GB therapy to exploit tumor glucose dependence, potentially slowing progression, enhancing treatment sensitivity, and modulating the tumor microenvironment. However, evidence is preliminary, with limited clinical trials and adherence challenges, highlighting the need for larger controlled studies [244,245,246].

### 10.2. Focused Ultrasound (FUS)

FUS is a noninvasive approach that transiently disrupts the blood–brain barrier to enhance drug delivery, immune infiltration, and tumor sensitivity to therapy in GB. While preclinical and early clinical studies are promising, challenges remain in optimizing targeting, minimizing off-target effects, and validating long-term safety and efficacy [247,248,249].

### 10.3. Nose-to-Brain Delivery

Nose-to-brain delivery offers a non-invasive route to bypass the blood–brain barrier, enabling the direct transport of therapeutic agents to the central nervous system via the olfactory and trigeminal pathways. Advances in nanocarriers, mucoadhesive formulations, and targeted ligands have improved drug stability, residence time, and tumor-specific accumulation, making this approach a promising strategy to enhance GB treatment efficacy [250,251,252].

### 10.4. NG101m

A trial is planned to test NG101m (given orally) in addition to the standard temozolomide + radiation treatment to see whether it improves 2-year overall survival [253].

### 10.5. Neoadjuvant and Adjuvant Dual Checkpoint Blockade

A trial combining ipilimumab + nivolumab (given both intravenously and intracranially) in recurrent resectable GB is being studied as a neoadjuvant + adjuvant approach [254].

### 10.6. Non-Coding RNA-Targeted Therapy and Drug Repurposing

Recent reviews highlight interest in targeting non-coding RNAs (e.g., microRNAs, lncRNAs) to modulate GB behavior (proliferation, invasion, therapy resistance). Repurposed agents such as disulfiram and aspirin have been considered in preclinical or early clinical settings [255]. Disulfiram has demonstrated the potential to overcome chemoresistance by inhibiting MGMT and inducing copper-dependent cytotoxicity, while aspirin is being explored for its role in inhibiting the COX-2/PGE2 pathway to modulate the inflammatory tumor microenvironment.

### 10.7. Optimization of Treatment Scheduling

Mathematical and computational modeling enables the optimization of GB treatment scheduling by simulating tumor dynamics and therapeutic responses to personalize and improve therapeutic outcomes. The algorithms generated can suggest alternative dosing schedules (e.g., extended intervals, modified fractionation). This might help improve outcomes or delay resistance in GB, though clinical validation is pending [256,257,258].

## 11. Comparative Synthesis of the Glioblastoma Translational Landscape

To provide a holistic view of the current GB landscape, the discussed modalities are synthesized in Table 2. This comparison explicitly positions standard-of-care interventions against emerging experimental frontiers, mapping the journey from bench to bedside.

Table Legends and Definitions

- *Evidence Quality*:
  ➢ **High**: Supported by randomized controlled trials (RCTs) or large-scale multicenter prospective studies.
  ➢ **Moderate**: Supported by Phase I/II trials, retrospective cohorts, or well-validated pilot studies.
  ➢ **Low**: Primarily supported by in vitro or in vivo animal models.
- *Typical Effect Size*: Refers to the relative impact on overall survival (OS), progression-free survival (PFS), or diagnostic accuracy as reported in the primary literature.
- *Clinical Readiness*: Ranges from standard of care (widely implemented) to pre-clinical (not yet approved for human use).
- *Translational Barrier*: The primary technical or biological hurdle preventing the modality from achieving higher efficacy or broader clinical adoption.

Table 2 serves as a multidimensional map of the current neuro-oncological toolkit. It highlights a critical paradox in glioblastoma research: the modalities with the highest effect size (such as Biomimetic Nanoformulations and Hippo pathway inhibitors) often possess the lowest clinical readiness due to the immense complexity of human physiology compared to laboratory models. Conversely, established “bedrock” therapies like hypofractionated radiotherapy and TTFields exhibit high evidence quality and are the current clinical standards. A significant emerging bridge is FUS; this modality is currently transitioning from clinical trials into routine practice by providing a non-invasive way to temporarily “open” the BBB, potentially increasing the effect size of other lower-readiness strategies like nanoparticles or immunotherapies. Finally, the inclusion of bioinformatics and machine learning illustrates the shift toward computational oncology, where “clinical readiness” is moving rapidly as models become more explainable and standardized across institutions.

## 12. Ongoing Clinical Trials on GB

The landscape of GB treatment is continuously evolving, with numerous clinical trials underway seeking to uncover more effective therapies [259]. The Perry trial (officially the CE.6 Phase III trial) established a new standard of care for elderly patients (aged 65 and older) with newly diagnosed glioblastoma. Led by Dr. James Perry and published in the New England Journal of Medicine in 2017, the study demonstrated that adding the chemotherapy drug temozolomide to a shortened course of radiation therapy significantly improves survival without compromising quality of life [260]. In a Nordic trial, a multicenter randomized trial focused on elderly patients with GB, the combination of female sex and MGMT promoter methylation served as a critical determinant of superior survival outcomes, highlighting the necessity of sex-based stratification when making clinical treatment decisions for glioblastoma patients [261].

### 12.1. Landmark Clinical Trials and Therapeutic Breakthroughs

Over the last six years, several promising studies have progressed through different stages of investigation:

▪ The INDIGO trial NCT04164901, a Phase 3 study spearheaded by the Dana-Farber Cancer Institute, is investigating the efficacy of vorasidenib in patients with Grade 2 gliomas exhibiting IDH1 or IDH2 mutations [262]. The Phase I trial (NCT02481154) evaluated vorasidenib, a brain-penetrant dual IDH1/2 inhibitor, in patients with recurrent or progressive mutant IDH glioma. The drug was well tolerated, with reversible liver enzyme elevations as the main toxicity. In non-enhancing gliomas, vorasidenib achieved an 18% response rate and a median progression-free survival of 36.8 months, showing promising antitumor activity [263].
▪ In another significant venture, UNC Health is conducting a Phase 2b clinical study (IGV-001) that examines a combination immunotherapy approach in newly diagnosed GB patients. This multicenter trial, aiming to enroll 93 participants, is poised to assess both the safety and efficacy of this novel therapeutic strategy. The trial underscores the increasing relevance of immunotherapy in the GB treatment paradigm [264].
▪ The University of California, San Diego (UCSD) is actively engaged in a diverse array of clinical trials targeting GB. These trials encompass a range of strategies, including the investigation of drug-resistant immunotherapy that combines activated gene-modified T cells with temozolomide, as well as studies evaluating the efficacy of agents like berubicin and enzastaurin in conjunction with temozolomide. This portfolio of trials at UCSD illustrates the multifaceted nature of current research efforts in GB treatment [265].
▪ Oncolytic virus therapy Teserpaturev (G47∆, “Delytact”) was approved in Japan for malignant glioma, including recurrent/residual GB and used via intratumoral injection. In clinical trials it showed an 84.2% one-year survival in a certain group, with a median overall survival of ~20.2 months [266].
▪ Furthermore, the role of immunotherapy in GB is being explored through various trials, with a significant emphasis on nivolumab, an anti-PD-1 antibody. Trials such as CheckMate 143 and NCT02550249 have explored its application in both recurrent and newly diagnosed GB. Despite some trials not achieving their primary endpoints, they highlight the intricacies of GB treatment and the necessity for continued research in this arena [267]. Table 3 exhibits the scenario of current worldwide trials of GB.

### 12.2. Lessons from the Frontier: Analyzing Landmark Trial Failures

The translational path for GB is paved with landmark Phase III failures that underscore the disease’s formidable resistance mechanisms. The **RTOG 0825** and **EORTC 26101** trials demonstrated that, while anti-angiogenic agents like Bevacizumab improve -PFS, they fail to extend OS due to adaptive shifts toward more invasive mesenchymal-like phenotypes [268,269]. Similarly, the **CheckMate series (143, 498, 548)** proved that PD-1 blockade alone is insufficient to overcome the ‘cold’ immunosuppressive landscape of GB, highlighting the need for the neoadjuvant and combinatorial strategies discussed in Section 5.1 [137,138]. Furthermore, the failure of the **ACT IV trial**—testing the Rindopepimut (EGFRvIII) vaccine—provided a critical lesson in antigen escape, where the selective pressure of the vaccine led to the outgrowth of EGFRvIII-negative clones [161]. Collectively, these results dictate that future frontiers must prioritize multifunctional nanoformulations and multi-antigen targeting to address the spatial and temporal heterogeneity that rendered these single-target interventions unsuccessful.

Despite decades of clinical investigation, the GB trial landscape continues to suffer from high failure rates, modest efficacy, and challenges in translating preclinical successes into clinical benefit. Heterogeneity, therapy resistance, and limited trial generalizability remain significant barriers, often compounded by small sample sizes and lack of external validation. While innovative strategies such as adaptive trial designs, biomarker-guided enrollment, and machine learning integration show potential, substantial progress will depend on large-scale multi-institutional collaboration and rigorous validation across diverse patient populations.

**Table 3:** Current ongoing clinical trial landscape for glioblastoma worldwide.

Trial Name	NCT ID	Phase	Primary Endpoint(s)	Recruitment Status (as of Q1 2026) Reference	Est. Completion
**GBM AGILE** (Platform Trial)	NCT03970447	II/III	Overall Survival (OS)	Active/Recruiting	December 2026
**IL13Rα2-CAR T cells** ± Checkpoint	NCT04003649	I	Safety/Dose-Limiting Toxicities (DLT)	Active/Recruiting	June 2027
**Chlorotoxin-domain CAR T** (CTX-CAR)	NCT04214392	I	Safety/MTD	Active/Recruiting	June 2026
**CART-EGFR-IL13Rα2** (Bivalent CAR)	NCT05168423	I	Safety/Feasibility	Active/Recruiting	October 2025 *
**[^177^Lu]Lu-DOTA-TATE** in GBM	NCT05109728	I	Safety/MTD	Active/Recruiting	July 2027
**Verteporfin Photodynamic Therapy**	NCT04590664	I/II	Safety/MTD	Active/Recruiting	August 2025 *
**Lerapolturev** (PVSRIPO)	NCT02986178	II	OS at 24 months	Completed	Completed February 2023
**VBI-1901** (Dendritic Cell Vaccine)	NCT03382977	I/II	Safety/Immunogenicity	Active/Recruiting	December 2026
**ReSPECT-GBM** (^186^Re Obisbemeda)	NCT01906385	I/II	Safety/MTD/PFS	Active/Recruiting	December 2025 *
**IL13Rα2 CAR** (New Structure)	NCT06355908	I	Safety/MTD/RP2D	Suspended	May 2027 **

The table outlines a cross-section of high-priority therapeutic investigations categorized by modality, including adaptive platform trials, cellular immunotherapies, and targeted radiopharmaceuticals. (A) Adaptive Designs: The GBM AGILE trial represents a shift toward “seamless” Phase II/III designs, allowing for the rapid evaluation of multiple arms against a common control to accelerate the discovery of signals for overall survival (OS). (B) Immunotherapeutic Frontiers: Several entries highlight the evolution of chimeric antigen receptor (CAR) T cell therapy, moving from monovalent targets (IL13Rα2) to bivalent structures (EGFR-IL13Rα2) and toxin-derived domains (Chlorotoxin) to address the hallmark problem of antigen escape and tumor heterogeneity. (C) Delivery and Precision Medicine: Trials such as ReSPECT-GBM and [^177^Lu]Lu-DOTA-TATE investigate the safety and Maximum Tolerable Dose (MTD) of localized radiopharmaceutical delivery, aiming to bypass the blood–brain barrier (BBB) and provide targeted cytotoxicity. (D) Metrics of Success: While early-phase (Phase I) trials focus on safety and Dose-Limiting Toxicities (DLT) to establish a Recommended Phase 2 Dose (RP2D), more advanced Phase II/III studies transition to efficacy endpoints such as progression-free survival (PFS) and OS. The recruitment status reflects the active nature of these investigations as of Q1 2026, including trials that remain open for long-term patient follow-up despite reaching primary completion. (Sources: [268,270,271,272,273,274,275,276,277,278,279]). Note: * Trial completed but remains open for longitudinal subject follow-up. ** Currently suspended.

## 13. The Future of GB Therapy: A Clinician’s Perspective

Modern neuro-oncological practice has evolved from a purely cytoreductive approach to a multi-modal synthesis of molecular diagnostics and precision-guided surgery. The utility of fluorescein and indocyanine green is now established as a Class I/II evidentiary standard for improving the extent of resection and progression-free survival by illuminating infiltrative tumor margins [280,281]. This visualization is increasingly augmented by targeted probes binding to VEGF, which serve as intraoperative biomarkers for the hypervascularity and metabolic aggressiveness codified in the 2021 WHO Classification of Tumors of the CNS [26,282].

Clinical data suggest a high concordance between intraoperative morphological features—such as friable neovascularization and central necrosis—and underlying molecular signatures [283,284,285]. These ‘macroscopic biomarkers’ frequently correlate with the high SR-B1 expression and lipid-metabolic reprogramming that drive GB invasiveness [3,286,287]. In this context, surgical resection serves as a prerequisite for the delivery of biomimetic nanoparticle platforms. These systems utilize a cholesterol mimicry strategy to exploit the tumor’s metabolic dependency on exogenous lipids, facilitating the SR-B1-mediated internalization of cytotoxic payloads while bypassing the BBB [3,75,76,77,286,287,288].

Furthermore, the synergy between metabolic interventions, such as statin therapy or ketogenic protocols and nanoparticle delivery represents a burgeoning area of ‘metabolic-precision’ crosstalk. By integrating real-time intraoperative data with longitudinal molecular monitoring, research suggests a pathway for clinicians to transition from palliative management toward a data-driven individualized therapeutic framework. While prospective evidence is still maturing, this convergence of surgical innovation, molecular profiling, and intelligent nanomedicine constitutes a potential shift toward addressing the spatial and temporal heterogeneity that has historically rendered GB resistant to traditional monotherapies.

### Concluding Remarks

Glioblastoma remains one of the most formidable challenges in oncology, with current therapeutic strategies offering only modest survival benefits. Advances in molecular diagnostics, radiomics, and liquid biopsy technologies are beginning to refine patient stratification, enabling earlier detection and more precise monitoring of disease progression. On the therapeutic front, emerging approaches such as immunotherapy, gene-targeted interventions, and nanotechnology-based systems are being actively investigated. Lipoprotein-based nanoparticles have shown promise in enhancing drug delivery across the blood–brain barrier; however, they are absent from most of the reviews, whereas FUS offers a noninvasive method to transiently disrupt this barrier and improve therapeutic penetration. Nose-to-brain delivery strategies further expand the armamentarium by bypassing systemic circulation to achieve more direct central nervous system targeting. Advanced MRI and radiomic imaging have become central to GB diagnostics, enabling noninvasive tumor characterization, early detection, and precise monitoring of treatment response, thereby improving patient stratification and guiding personalized therapy. Despite these advances, tumor heterogeneity, adaptive resistance, and the lack of multi-institutional validation continue to hinder durable clinical translation.

As our understanding of the molecular and genetic complexity of GB advances, integrating genomic, histologic, and microenvironmental data is essential for rational clinical trial design. A 2020 Society for Neuro-Oncology Think Tank highlighted key barriers, including restrictive eligibility criteria, limited control arms in Phase 2 trials, and a lack of positive Phase 2 data before Phase 3 initiation [289]. Adaptive platforms like GB AGILE (NCT03970447), along with umbrella and basket trial designs, aim to overcome these challenges and improve trial efficiency.

Moving forward, harmonized data sharing, collaborative trial frameworks, and multimodal precision-driven strategies will be essential to advance diagnostics and therapeutics for patients with GB.

## Figures and Tables

**Table 1:** Pivotal genetic changes/biomarkers in different types of gliomas classified by the WHO and their CNS WHO grades.

Tumor Type	Altered Molecular Profiles/Genes	CNS WHO Grade	Key Clinical/Diagnostic Change (WHO 2026, 6th Ed. [28])
***Adult-type diffuse gliomas***			
Glioblastoma, IDH-wildtype	IDH-wild type, TERT promoter, chromosomes 7/10, EGFR	4	Diagnosis no longer requires histological necrosis if molecular markers are present.
Astrocytoma, IDH-mutant	IDH1, IDH2, CDKN2A/B	2,3,4	Presence of *CDKN2A/B* homozygous deletion now mandates Grade 4 status.
Oligodendroglioma, IDH-mutant, and 1p/19q-codeleted	IDH1, IDH2, 1p/19q, TERT promoter, CIC, FUBP1, NOTCH1	2,3	“Anaplastic” nomenclature fully retired in. Graded based on mitotic activity and necrosis.
***Pediatric-type diffuse low grade gliomas***			
Diffuse low-grade glioma, MAPK pathway-altered	FGFR1, BRAF	N/A	
Diffuse astrocytoma, MYB- or MYBL1-altered	MYB, MYBL1	1	
Angiocentric glioma	MYB	1	
Polymorphous low-grade neuroepithelial tumor of the young	BRAF, FGFR family	1	
***Pediatric-type diffuse high grade gliomas***			
Diffuse midline glioma, H3 K27-altered	H3 K27, TP53, ACVR1, PDGFRA, EGFR, EZHIP	4	Expanded to include EZHIP overexpression as a defining molecular feature.
Diffuse hemispheric glioma, H3 G34-mutant	H3 G34, TP53, ATRX	4	
Diffuse pediatric-type high-grade glioma, H3-wildtype, and IDH-wildtype	IDH-wildtype, H3-wildtype, PDGFRA, MYCN, EGFR (methylome)	4	
Infant-type hemispheric glioma	NTRK family, ALK, ROS, MET	N/A	
***Circumscribed astrocytic gliomas***			
Pilocytic astrocytoma	KIAA1549-BRAF, BRAF, NF1	1	
High-grade astrocytoma with piloid features	BRAF, NF1, ATRX, CDKN2A/B (methylome)	4	HGAP is now a formally recognized molecular entity (Grade 4).
Pleomorphic xanthoastrocytoma	BRAF, CDKN2A/B	2,3	
Subependymal giant cell astrocytoma	TSC1, TSC2	1	
Chordoid glioma	PRKCA	2	
Astroblastoma, MN1-altered	MN1	N/A	
***Ependymal glioma tumors***			
Supratentorial ependymomas	ZFTA, RELA, YAP1, MAML2	1,2,3	The nomenclature has shifted fully from “RELA-fusion” to ZFTA-fusion.
Posterior fossa ependymomas	H3 K27me3, EZHIP (methylome)	1,2,3	
Spinal ependymomas	NF2, MYCN	1,2,3	
Spinal ependymoma (SP-EPN)			
Spinal ependymoma with MYCN mutation (SP-MYCN)	MYCN		
Myxopapillary ependymoma (MPE)	Chromosomal abnormalities, DNA methylation	2	Now classified as Grade 2 due to their clinical recurrence rates (changed from the older Grade 1 designation).
Subependymoma (SE)	DNA methylation, TERT promotor mutation, loss of chromosome 6	1,2	
spinal ependymoma NEC/NOS	No distinct biomarker yet		

Note: The WHO 2021 (5th ed.) [26,27] classification updated to reflect the WHO CNS 6th Edition (2026) [28] framework as of April 2026, incorporating revised molecular grading criteria for gliomas and formalized nomenclature for pediatric-type diffuse gliomas.

**Table 2:** Multidimensional Assessment of Glioblastoma Management Strategies: From Standard of Care to Pre-clinical Innovations.

Modality	Evidence Quality	Typical Effect Size	Clinical Readiness	Translational Barrier
*Surgical and Radiotherapies*	High	Moderate to High	Standard of Care	Infiltrative recurrence
*High-Resolution Mapping (fMRI/DTI)*	High	Moderate (Safety)	Routine Clinical	Intraoperative brain shift
*TTFields (Optune)*	High	Moderate (OS Benefit)	Standard of Care	Patient compliance/cost
*Biomarker Targeting (MGMT)*	High	High (Prognostic)	Routine Clinical	Tumor heterogeneity
*Advanced MRI (Perfusion/Spec)*	Moderate/High	Moderate (Diagnostic)	Routine Clinical	Complex post-processing
*Focused Ultrasound (FUS)*	Moderate	High (Targeting)	Clinical Trials	Scalability/skull density
*Bioinformatics/Machine Learning*	Moderate	High (Accuracy)	Early Clinical	Model interpretability
*Immunotherapies*	Moderate	Low to Variable	Clinical Trials	Immunosuppressive tumor microenvironment (TME)
*Radiomics/Radiogenomics*	Moderate	Moderate	Research/Pilot	Lack of standardization
*Hippo Pathway Targeting*	Low	High (Mechanistic)	Pre-Clinical	Target specificity
*Liquid Biopsy*	Low/Moderate	Low (Sensitivity)	Experimental	Blood–brain barrier (BBB)
*Biomimetic Nanoformulations*	Low	High (In Vivo)	Pre-Clinical	Regulatory hurdles
*Stem Cells/Extracellular Vesicles (EVs)*	Low	Moderate	Pre-Clinical	Delivery and persistence

## Data Availability

No new data were created or analyzed in this study. Data sharing is not applicable to this article.

## Abbreviations

ACVR1	Activin A Receptor Type I
ADAMTS6	A Disintegrin And Metalloproteinase with Thrombospondin Motifs 6
AKT	Protein Kinase B (PKB)—often referred to by its gene name AKT
ALK	Anaplastic Lymphoma Kinase
ATRX	Alpha Thalassemia/Mental Retardation Syndrome X-Linked
AUC	Area Under the Curve
BBB	Blood–Brain Barrier
BTB	Blood–Tumor Barrier
BNFs	Biomimetic Nano-Formulations
BNCT	Boron Neutron Capture Therapy
BRAF	B-Raf Proto-Oncogene
CACNA2D1	Calcium Voltage-Gated Channel Auxiliary Subunit α2Δ1
CAPN6	Calpain 6 (Calcium-Dependent Cysteine Protease 6)
CAP	Cold Atmospheric Plasma
CARs	Chimeric Antigen Receptors
CD47	Cluster of Differentiation 47
CDKN2A/B	Cyclin-Dependent Kinase Inhibitor 2A and 2B
CSF	Cerebro Spinal Fluid
CIC	Capicua Transcriptional Repressor (downstream effector in RTK/MAPK signaling)
CIRT	Carbon Ion Radiotherapy
CNS	Central Nervous System
COX-2	Cyclooxygenase-2
CRISPR	Clustered Regularly Interspaced Short Palindromic Repeats
CSCs	Cancer Stem Cells
CSF	Cerebrospinal Fluid
CT	Computed Tomography
cfDM	Cell-Free DNA Methylation
ctDNA	Circulating DNA
CTCs	Circulating Tumor Cells
ctRNA	Circulating Tumor RNA
CTLA-4	Cytotoxic T-Lymphocyte-Associated Protein 4
ctDNA	Circulating Tumor DNA
DCs	Dendritic Cells
DTI	Diffusion Tensor Imaging
ECM	Extracellular Matrix
EphA2	Ephrin Type-A Receptor 2
EGFR	Epidermal Growth Factor Receptor
EOR	Extent of Resection
ER	Extracellular Signal-Regulated Kinase
EVs	Extracellular Vesicles
EZHIP	Enhancer of Zeste Homologs Inhibitory Protein
FGFR	Fibroblast Growth Factor Receptor
fMRI	Functional Magnetic Resonance Imaging
FUBP	Far Upstream Element (FUSE) Binding Protein
FUS	Focused Ultrasound
GB	Glioblastoma
GBM	Glioblastoma Multiforme
GD2	Ganglioside 2
GDF15	Growth Differentiation Factor 15
GLP	Good Laboratory Practice
GP130	Glycoprotein 130
GSCs	Glioma Stem Cells
HDL	High-Density Lipoprotein
HER2	Human Epidermal Growth Factor Receptor 1
HLA	Human Leukocyte Antigen
ICIs	Immune Checkpoint Inhibitors
IDH	Isocitrate Dehydrogenase
IDHwt	Isocitrate Dehydrogenase Wild Type
IKBKE	Inhibitor of Nuclear Factor Kappa-B Kinase Subunit Epsilon
IL-10	Interleukin-10
IL-13R⍺2	Interleukin 13 receptor, Alpha 1
KIAA1549	KIAA1549 Gene
KD	Ketogenic Diet
LAG3	Lymphocyte Activation Gene 3
LATS1/2	Large Tumor Suppressor Kinase 1 and 2
LDL	Low-Density Lipoprotein
LDLR	Low-Density Lipoprotein Receptor
ML	Machine Learning
MAML2	Mastermind-Like Transcriptional Coactivator 2
MAPK	Mitogen-Activated Protein Kinase
MEK	Mitogen-Activated Protein Kinase (also known as MAP2K)
MCL-1	Myeloid Cell Leukemia Protein 1
MDSCs	Myeloid-Derived Suppressor Cells
MDM2	Mouse Double Minute 2
MET	MET Proto-Oncogene
MN1	Meningioma 1 Gene
MGMT	O6-methylguanine-DNA Methyltransferase
miRNAs	Micro Ribonucleic Acid
MPS	Mononuclear Phagocyte System
MRI	Magnetic Resonance Imaging
MSCs	Mesenchymal Stem/Stromal Cells
mTOR	Mammalian (or mechanistic) Target of Rapamycin
MYB	Myeloblastosis
MYCN	v-Myc Avian Myelocytomatosis
NCCN	National Comprehensive Cancer Network
NODDI	Neurite Orientation Dispersion and Density Imaging
NF1	Neurofibromin 1
NK cell	Natural Killer Cells
NFKB	Nuclear Factor Kappa-Light-Chain-Enhancer of Activated B Cells
NKT	Natural Killer T cells
NOTCH	Notch Receptor Family
NP	Nanoparticle
NSCs	Neural Stem Cells
NTRK family	Neurotrophic Tyrosine Receptor Kinase Family
OS	Overall Survival
OV	Oncolytic Virotherapy
p53	Tumor Protein p53
PCSK9	Proprotein Convertase Subtil-isin/kexin Type 9
PD-1	Programmed Cell Death Protein 1
PDCD4	Programmed Cell Death 4
PD-L1	Programmed Death-Ligand 1
PDGFRA	Platelet-Derived Growth Factor Receptor Alpha
PGE2	Prostaglandin E2
PI3K	Phosphoinositide 3-kinase
PLGA	Poly(lactic-co-glycolic) Acid
PT	Proton Therapy
PTEN	Phosphatase and Tensin Homolog
RAF/MEK/ERK	The MAPK Cascade
RAF	Rapidly Accelerated Fibrosarcoma
RB	Retinoblastoma
RAS	Rat Sarcoma Virus
RELA	v-Rel Avian Reticuloendotheliosis Viral Oncogene Homolog A
ReSPOND	Report, Evaluate, Stabilize, Preserve, Organize, Normalize, Document/Debrief
rHDL	Reconstituted High-Density Lipoprotein
RIT	Radioimmunotherapy
ROS	Ras Oncogene from Esteosarcoma
ROC	Receiver Operating Characteristic curve
RT	Radiotherapy
RTK	Receptor Tyrosine Kinase
SAA1	Serum Amyloid A1
SAA2	Serum Amyloid A2
scRNA-seq	Single-Cell RNA Sequencing
SR-B1	Scavenger Receptor Class B type 1
siRNAs	Small Interfering RNAs
SHH	Sonic Hedgehog
SOC	Standard of Care
SRS	Stereotactic Radiosurgery
STING	Stimulator of Interferon Genes
TAAs	Tumor-Associated Antigens
TAMs	Tumor-Associated Macrophages
TAZ	Transcriptional Co-Activator with PDZ-binding Motif
TCIA	The Cancer Imaging Archive
TEPs	Tumor-Educated Platelets
TERT	Telomerase Reverse Transcriptase
TGF-ß	Transforming Growth Factor-Beta
TIE1	Tyrosine Kinase with Immunoglobulin-Like and EGF-Like Domains 1
TIGIT	T Cell Immunoreceptor with Ig and ITIM Domains
TMZ	Temozolomide
TP53	Tumor Protein p53
Tregs	Regulatory T Cells
TSAs	Tumor-Specific Antigens
TSC1/2	Tuberous Sclerosis Complex 1 and 2
TTFields	Tumor-Treating Fields
USP26	Ubiquitin Specific Peptidase 26
US FDA	United States Food and Drug Administration
WNT	Wingless/Integrated Signaling Pathway
WHO	World Health Organization
YAP1	Yes-Associated Protein 1
ZFTA	Zinc Finger Transcription Factor AT-Hook 1
